# High-resolution investigations of fault architecture in space and time

**DOI:** 10.1038/s41598-025-86104-w

**Published:** 2025-01-17

**Authors:** Leonardo Del Sole, Giulio Viola, Luca Aldega, Vincenzo Moretto, Manuel Curzi, Ruikai Xie, Luigi Cantelli, Gianluca Vignaroli

**Affiliations:** 1 Dipartimento di Scienze Biologiche, Geologiche ed Ambientali, Università di Bologna, Bologna, Italy; 2 Dipartimento di Scienze della Terra, Sapienza Università di Roma, Rome, Italy; 3 Geological Survey of Norway, Trondheim, Norway

**Keywords:** Tectonics, Structural geology, Mineralogy

## Abstract

Heterogeneous fault architecture affects crustal seismotectonics and fluid migration. When studying it, we commonly rely on static conceptual models that generally overlook the absolute time dimension of fault (re)activation. Heterogenous faults, however, represent the end-result of protracted, cumulative and intricate deformation histories. This may lead to inaccurate reconstructions of tectonic histories and flowed models of fault hydro-mechanical behavior. We adopt here a multitechnique approach building upon the examination of now juxtaposed but not coeval *brittle structural facies* (BSF), which offer multiscalar insights in the spatio-temporal-thermal fault evolution. Our approach is applied to the Carboneras Fault, unveiling a ~ 25 Myr-long polyphase structural and thermal evolution. This led to a complex fault architecture, where BSFs exhibit a stark heterogeneity in fault rock and permeability, ultimately generating very different space- and time-dependent fault hydro-mechanical behaviors. Therefore, fault architectures shall be seen as dynamic features from which to extrapolate time-integrated comprehensive fault models accounting for the entire deformed rock volume and fault life span. We demonstrate that high-resolution studies of fault architectures are required to elucidate modes of fault growth and evolution, decipher long-lived, polyphase tectonic and thermal histories, and understand the influence of heterogenous fault architecture on hydraulic compartmentalization and earthquake rupture dynamics.

**Supplementary Information:**

The online version contains supplementary material available at 10.1038/s41598-025-86104-w.

## Introduction

Geological descriptions of faults commonly encompass highly localized displacements on one or more discrete slip planes or more spatially distributed deformation in tabular or anastomosing and architecturally complex zones. Complex and heterogenous fault geometries and architectures have been shown to exert a significant role on fault mechanical stability^1–3^ and to steer earthquake behavior^4–6^ and permeability structure^7–9^, thus influencing seismotectonic styles and fluid flow and compartmentalization in the brittle crust.

Fault architectural complexity is commonly associated with long-lived and multiply reactivated (mature) faults, their complexity further increasing by each new accommodated faulting episode^10–12^. Grasping the absolute timing of faulting, which can span dozens of million years^12–14^, is, in this perspective, key to the unveiling of the time-integrated hydro-mechanical fault behavior, because multiple deformation episodes repeatedly change the bulk and local petrophysical and rheological properties of the deforming rock volume^7,8,15,16^. Additionally, the interplay between fluid ingress and circulation and mechanical rupturing can lead to complex and transient slip behaviors^6,17–20^. However, due to the common structural intricacies of faults and the analytical challenges associated with the geochronology of synkinematic minerals^21–25^, the time dimension of deformation and continuous evolution of complex geometrical and hydro-mechanical properties during fault growth is still poorly investigated and understood, being usually overlooked. Consequently, conceptual evolutionary models of mature faults tend to simplify their internal architecture by neglecting the detection and characterization of the many faulting episodes that may be recorded by and within the very same fault architecture as well as by ignoring or downplaying the involved time dimension of the faulting history. This, in turn, hampers our ability to distinguish and interpret now spatially juxtaposed but not necessarily coeval or genetically correlated brittle structural facies (BSF)^11^. Indeed, mature faults may be structurally so complex to become extremely difficult to decipher and to reconcile with the much simpler conceptual and experimental fault evolutionary models that the scientific community generally relies on.

**Fig. 1:**
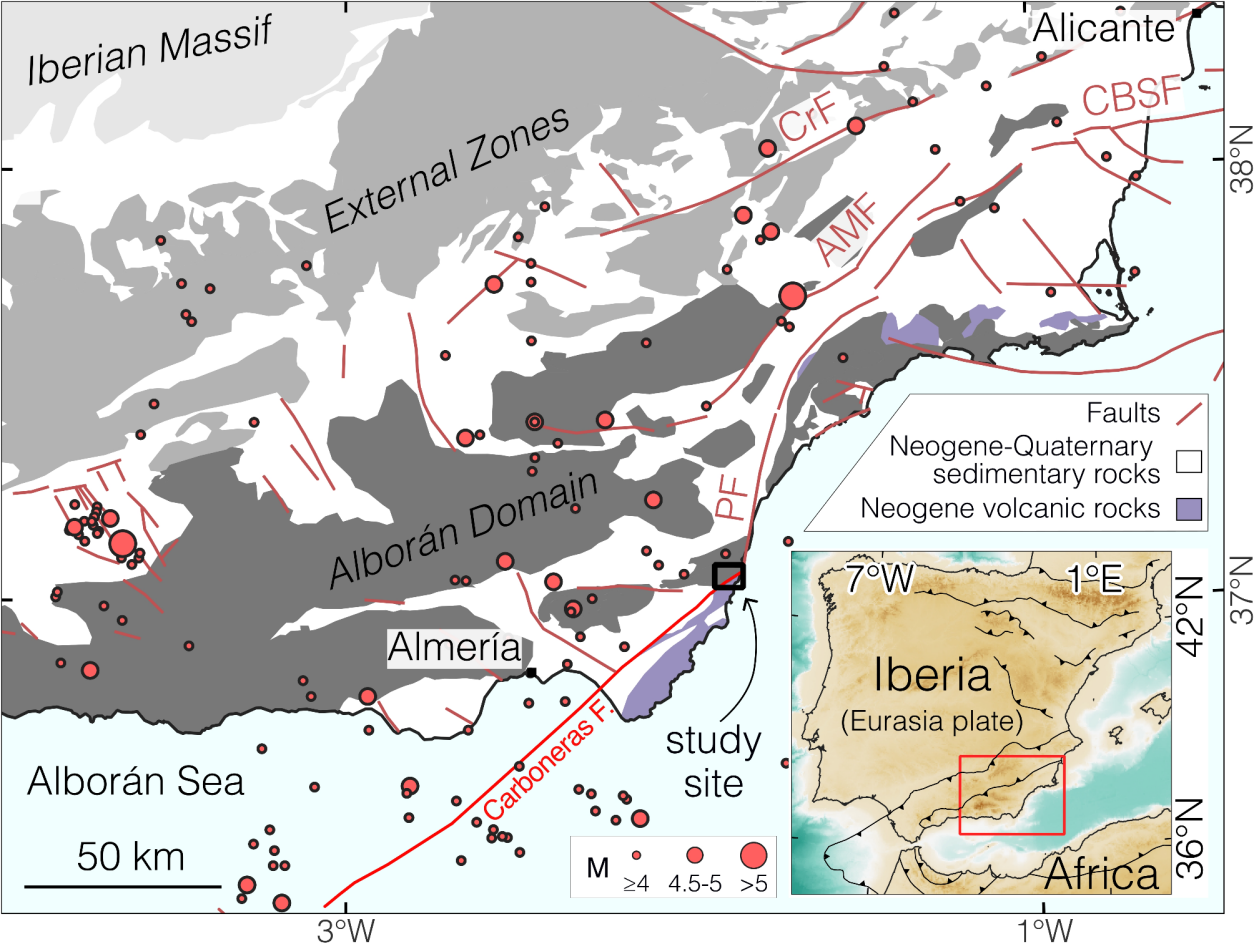
Location map. Simplified tectonic map of the central-eastern Betics (partly based on ref.^33^) in the western Mediterranean [bottom right inset: ETOPO 2022 relief model^34^and GSHHG shoreline Database v2.3.7^35^]. Seismogenic crustal faults are drawn after Basili et al.^36^. PF – Palomares Fault; AMF – Alhama de Murcia Fault; CBSF – Carrascoy - Bajo Segura Fault; CrF – Crevillente Fault. Seismicity (M ≥ 4) with depths ranging from 0 to 20 km during the period from April 1924 to April 2024^37^.

Aiming at improving our understanding of complex faults, we combined high-resolution field structural analysis with permeability determinations of various BSF’s and in-depth microanalytical characterizations of fault rocks, including optical microscopy, X-ray diffraction and K-Ar dating of synkinematic illite, to unravel the long-term spatiotemporal and temperature evolution of faulting along the Carboneras Fault (CF, Spain). The NE-SW-striking CF is a c. 140 km long, active, left-lateral strike-slip fault system in the Betic Cordillera, which is inferred to have accommodated c. 40 km of cumulated displacement since the Miocene^26–30^ (Fig. 1; refer to Supplementary Note 1 for further information on the geological framework of the CF). We show that it accommodated multiple episodes of on- and off-fault deformation and fluid ingress over a > 20 Myr-long polyphase faulting history at progressively colder temperature conditions (from > 275 to 70 °C), and that it responded to the interaction with inherited structural discontinuities and selective fault (de)activation to produce a stark heterogeneity in fault rock (with domains suggesting seismic slip, while others aseismic slip) and permeability (up to 4 orders of magnitude).

The presented study case is pivotal, from a methodological perspective of general validity, to establish and validate a versatile multi-technique approach that is well suited to unravel intricate deformation histories archived in any active or fossil fault. This approach is applicable in any structural setting and, as such, is instrumental to the building of ever more accurate fault zone conceptual and physical models based on high-resolution deterministic constraints. These improved models will benefit refined numerical and analogue simulations as well as experimental deformation studies aimed at predicting fault hydro-mechanical behavior in general^18,19^ but also in an applied perspective when considering the importance of faults and of their petrophysical properties to a variety of industrial activities (e.g., H_2_/CO_2_injection, waste disposal, production of geothermal and hydrocarbon resources)^31,32^, many of which are key to the success of the much needed green transition.

## Results

### Fault zone architecture and permeability structure

**Fig. 2:**
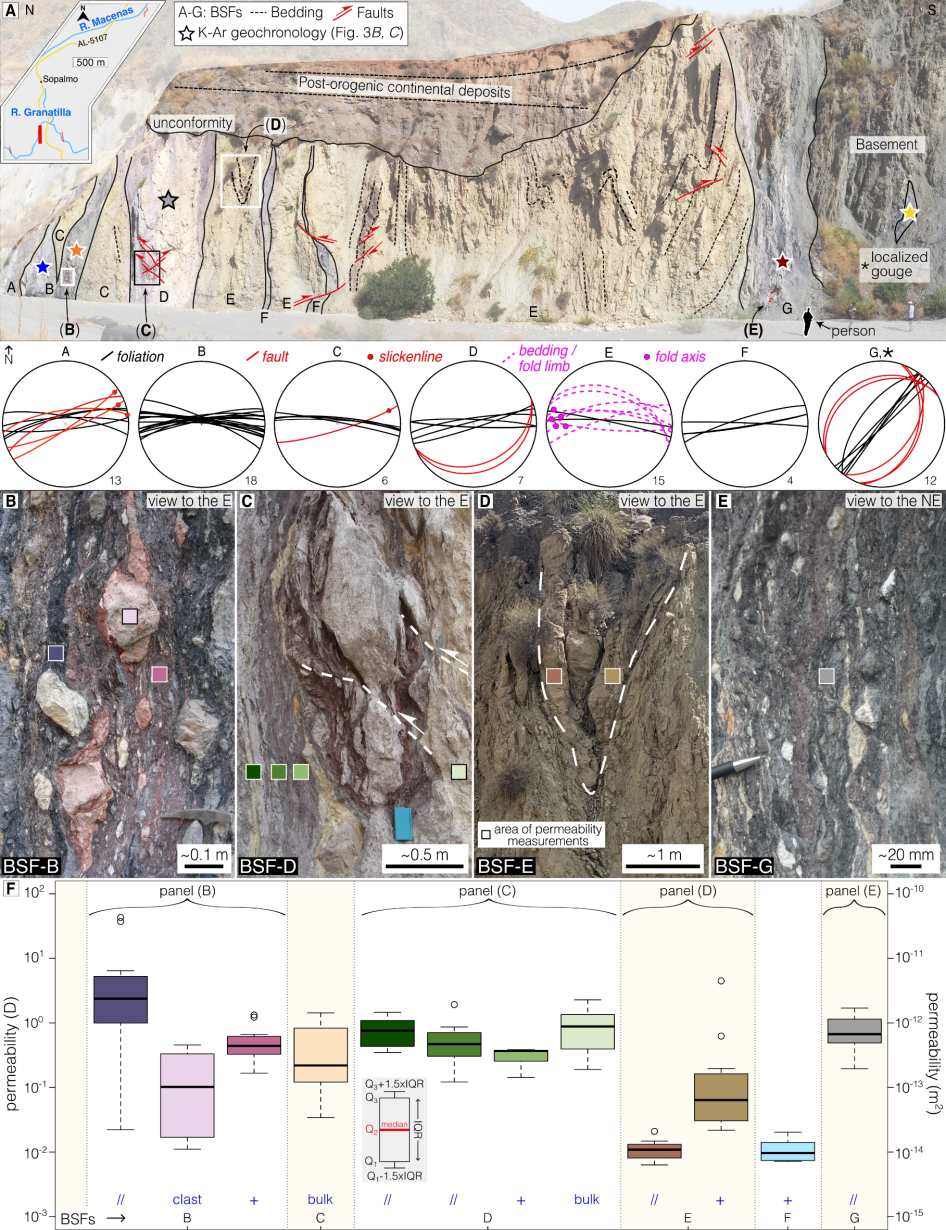
Structural and permeability data in RGA. (**a**) Panoramic view of RGA with geological-structural interpretation (see Supplementary Fig. 1 for uninterpreted figure). Stereonets are lower-hemisphere equal-area projections of faults, foliations, bedding planes, and fold limbs. (**b-d**) Field photos of key deformation structures: E-W trending fabrics including clasts embedded and flattened within foliated gouges, upright tight to isoclinal folds in layered sedimentary rocks and low-angle reverse faults. We interpreted these fabrics as multiple lines of evidence for flattening and shortening orthogonal the strike of the main foliation. (**e**) Steeply dipping NE-SW foliation crosscut by NE-SW low-angle reverse faults. (**f**) Log-scale permeability profile. Measurements were perfomed parallel (//) and orthogonal (+) to the foliation/bedding, in the unfoliated matrix (bulk) and in the embedded clasts. See Methods for further details and Supplementary Method 1 for boxplot construction.

In the following, we provide a close up on the CF architecture as exposed at a spectacular outcrop along the Rio Granatilla (RGA; Fig. 2a). Descriptions of the outcrop and its structures are provided below with further details in Supplementary Fig. 1.

*Outcrop RGA* (37°03’01"N, 1°52’53"W) is a c. 100 m long section composed of seven tightly juxtaposed BSFs (labelled A to G; Fig. 2a and Supplementary Fig. 1). RGA contains variably deformed, polychromatic, well foliated gouges embedding variably sized pelitic and psammitic clasts, a broad spectrum of sedimentary rocks generally preserving the original bedding (marl, siltstone, sandstone, conglomerate; BSF-C and -E), and volcaniclastic rocks mostly as transposed and dispersed blocks (BSF-C and -G; Supplementary Fig. 1). BSFs A through to F are characterized by a subvertical E-W pervasive and anastomosing foliation that embeds flattened clasts and squeezed upright tight to isoclinal folds (Fig. 2a–d). Faint evidence of strike-slip kinematics (abrasion striae with pitch > 170° or < 20°) is observed along the E-W to ENE-WSW foliation planes (Supplementary Fig. 1). BSF-G is geometrically remarkably different from all other BSFs as it is characterized by a NE-SW-striking (N040°) steeply dipping foliation that cuts across the E-W trending fabric (Fig. 2a,e and Supplementary Fig. 1). A localized black gouge is found within basement schist immediately to the south of BSF-G, with which it shares the same NE-SW high-angle fabric. In all BSFs, ENE-WSW to NE-SW striking, relatively late and low-angle reverse shear bands (often in conjugate pairs) crosscut both the E-W and NE-SW high-angle foliation planes (Fig. 2a,c,e and Supplementary Fig. 1).

A total of 117 in situ air permeability measurements were collected (Fig. 2f and Supplementary Table 1). Overall permeability ranges between 6.2 × 10^−15^ and 6.4 × 10^−12^ m^2^ (up to 4.2 × 10^−11^ m^2^ considering outliers), with a median of 3.6 × 10^−13^ m^2^ and an average of 5.9 × 10^−13^ m^2^. Results indicate a highly heterogenous and anisotropic permeability with up to 3 orders of magnitude (up to 4 considering outliers) of differences between adjacent BSFs.

### Fault gouge mineral assemblage

**Fig. 3:**
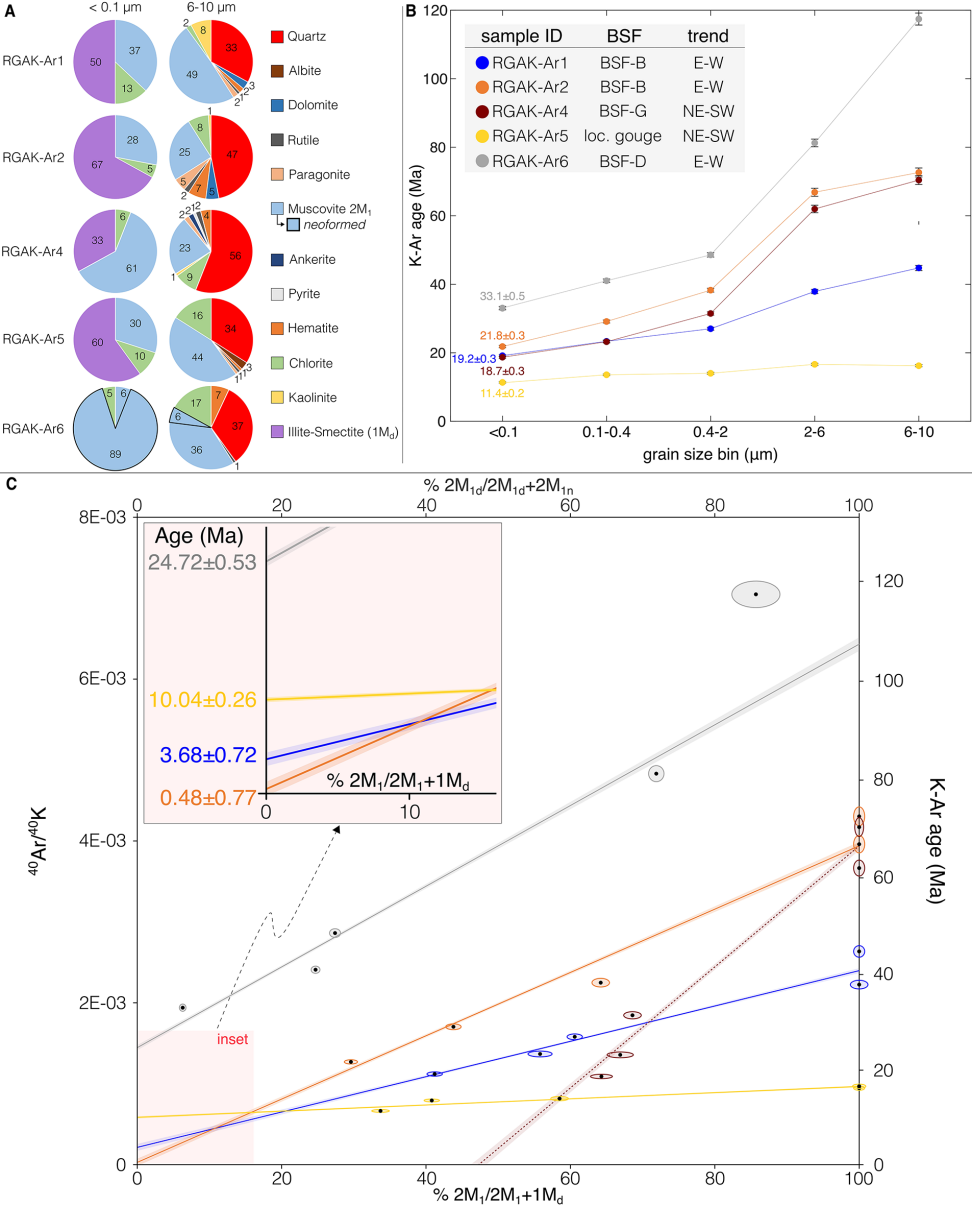
Mineral assemblage, K-Ar age and Illite Age Analysis (IAA) for the clay gouges of the CF. (**a**) XRD quantitative analysis (%wt) of the dated finest (< 0.1 μm) and coarsest (6–10 μm) grain size fractions of each gouge sample and illite polytype determination. Pie charts for all grain size fractions are in Supplementary Fig. 2. All XRD data are reported in Supplementary Table 2, while X-ray pattern raw files are stored in the figshare repository doi: 10.6084/m9.figshare.27055180.v3. (**b**) K-Ar age vs. grain size spectra. Error bars that are not visible are smaller than the symbol size. K-Ar raw data can be found in Supplementary Table 3, as well as in the figshare repository doi: 10.6084/m9.figshare.27055180.v3. (**c**) ^40^Ar/^40^K ratios are plotted against percentage of illite-2M_1_ (normalized to 100%), except for sample RGAK-Ar6 (grey) where ^40^Ar/^40^K ratios are plotted against percentage of muscovite‐2M_1_ (top x-axis). In this sample, we distinguished between detrital (2M_1d_) and neoformed (2M_1n_) muscovite (Supplementary Fig. 6). The ages of the authigenic illite‐1M_d_ (or muscovite-2M_1n_) end member (see inset; i.e., the age of the last faulting event) is estimated by linearly extrapolating to 0% illite‐2M_1_ (or muscovite-2M_1d_) and converting ^40^Ar/^40^K intercepts to K-Ar ages using the decay constants (see Methods and Supplementary Fig. 7). K‐Ar ages extrapolated to 100% illite‐2M_1_ (or muscovite-2M_1d_) represent a detrital component inherited from the host rock or correspond to the age of possible older tectonometamorphic events.

Twenty-five XRD analyses (Fig. 3 and Supplementary Data 1 and Supplementary Fig. 2) of five structurally constrained fault gouges (summarized in Fig. 3; their location is indicated in Fig. 2a and Supplementary Fig. 3, while their microtexture is shown in Supplementary Fig. 4) subdivided into five grain size fractions (< 0.1, 0.1–0.4, 0.4–2, 2–6 and 6–10 μm) allowed us to reconstruct the distribution of synkinematic and detrital minerals within the BSFs, to determine their polytypism and to constrain their formation temperature (see Methods). Gouge samples are a mixture of detrital minerals inherited from the host rock and synkinematic minerals (mostly mixed layers illite-smectite-1M_d_; see Supplementary Fig. 5 for documentation on illite crystal morphology) formed during fault slip and/or from fluids circulating within the fault zone.

In the following, for the sake of brevity, we will describe only the mineral assemblage of the coarsest (6–10 μm) and finest (< 0.1 μm) fractions (Fig. 3a; further details in Supplementary Data 1 and Supplementary Fig. 2). RGAK-Ar1 and RGAK-Ar2 contain muscovite-2M_1_ (25–49%), quartz (33–47%), dolomite (3–5%), hematite (2–7%), rutile (1–2%), paragonite (2–5%), chlorite (2–8%) and kaolinite (1–8%) in the 6–10 μm fraction that progressively decrease their content or disappear in the < 0.1 μm fraction (Fig. 3a). The < 0.1 μm fraction consists of high expandable R0 illite-smectite (50%), muscovite-2M_1_ (37%) and chlorite (13%) for RGAK-Ar1 and is made up of R1 illite-smectite (67%), muscovite-2M_1_ (28%) and chlorite (5%) for RGAK-Ar2 (Fig. 3a). RGAK-Ar6 contains a mineral assemblage composed of quartz, hematite, rutile, chlorite and inherited and neoformed muscovite-2M_1_ (Fig. 3a). These become progressively less abundant in the finer fractions where a general increase of neoformed muscovite-2M_1_ is instead observed, reaching 89% in the < 0.1 μm fraction. RGAK-Ar4 is mostly made up of quartz (56%), muscovite-2M_1_ (23%) and chlorite (9%) and subordinate amounts of ankerite, hematite, rutile, pyrite, paragonite and kaolinite not exceeding 4% in the 6–10 μm fraction. Synkinematic minerals (R0 illite-smectite with an illite content of 30%) reach 33% in the < 0.1 μm fraction. RGAK-Ar5 contains muscovite-2M_1_ (44%), quartz (34%), chlorite (16%), albite (3%), hematite (1%), paragonite (1%) and rutile (1%) in the 6–10 μm fraction. Synkinematic minerals are R1 illite-smectite with an illite content of 80% that reaches 60% in the < 0.1 μm fraction, whereas inherited non-clay minerals disappear.

### Fault gouge K-Ar geochronology

Aiming at constraining the age of the last slip event recorded in the BSFs and defining the absolute time dimension of CF faulting, we dated five fault gouges by means of K-Ar geochronology (see Methods). As shown in the cumulative “age vs. grain-size” plot (Fig. 3b), five separated grain size fractions were successfully dated for each gouge, with the obtained dataset invariably exhibiting a grain size-age correlation wherein the coarser the size fraction, the older the age. One sample (RGAK-Ar5), however, is characterized by a flat “age vs. grain-size” relationship, whereby the ages of all fractions are basically statistically identical irrespective of the dated size fraction. This suggests the dating of one generation of synkinematic illite in the fine fractions, which points to substantial authigenesis of K-bearing minerals during faulting^10,12^, providing a robust time constraint on the last recorded episode of slip. In multiple grain size fraction K-Ar dating, the finest fraction is taken as representing the last recorded increment of illite growth^12,24^. The finest fractions (< 0.1 µm) yield K-Ar dates of 19.2 ± 0.3 Ma (RGAK-Ar1), 21.8 ± 0.3 Ma (RGAK-Ar2), 18.7 ± 0.3 Ma (RGAK-Ar4), 11.4 ± 0.2 Ma (RGAK-Ar5) and 33.1 ± 0.5 Ma (RGAK-Ar6; Fig. 3b). These dates need to be considered as maximum ages of the last increment of slip in the respective gouges, because the dated K-bearing mineral phases may still be part of a mixture of synkinematic and detrital minerals inherited from the host rock during faulting^11,13^. We refined our K-Ar ages through the Illite Age Analysis (IAA) approach (see Methods; Fig. 3c) to account for the effects of host rock contamination, as attested by the presence of detrital illite or muscovite (Fig. 3a). The K-Ar IAA ages of fault gouges (Fig. 3c) highlight three statistically distinct faulting events during the (i) Chattian (24.72 ± 0.53 Ma), as recorded by an E-W trending BSF (RGAK-Ar6), (ii) Late Miocene (10.04 ± 0.26 Ma) along a NE-SW BSF (RGAK-Ar5) and (iii) Early Pliocene-Middle Pleistocene (between 3.68 ± 0.72 and 0.48 ± 0.77 Ma) along E-W BSFs (RGAK-Ar1 and RGAK-Ar2).

### Space-time-temperature evolution of the Carboneras Fault

The complex internal architecture of the CF as determined inhere reflects the juxtaposition of tabular to lensoidal BSFs. They are characterized by (i) an E-W-striking, subvertical pervasive foliation and upright folds, (ii) NNE-SSW to E-W reverse faults, and (iii) minor NE-SW strike-slip faults and steeply dipping foliation planes (BSF-G) which truncate older BSFs (BSFs A to F) and that are parallel to the CF regional trend. The documented tectonic fabrics and BSF contacts basically all strike E-W (with the exception of BSF-G), suggesting that *c.* N-S shortening led to the structural architecture in the entire study area within and around the CF (Fig. 2 and Supplementary Fig. 1).

**Fig. 4:**
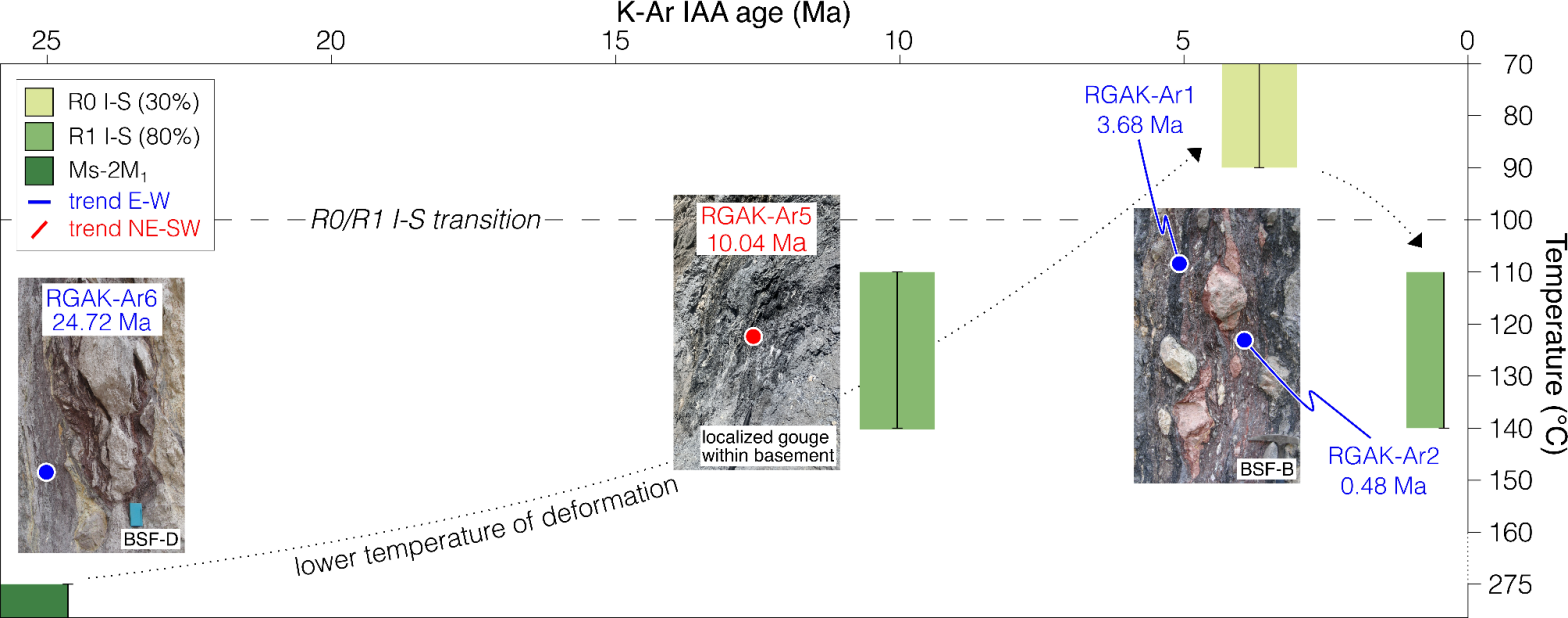
Space-time-temperature evolution of the CF. Temperature of synkinematic minerals vs. K-Ar IAA ages showing the long-lived fault activity of the CF at progressively colder conditions.

The oldest dated faulting event results from *c.* NNW-SSE directed, Chattian (24.72 ± 0.53 Ma) shortening. The occurrence of synkinematic muscovite-2M_1_in the finest dated fraction of fault gouge RGAK-Ar6 indicates that, during this shortening phase, the crystallization temperature was > 275 °C^38^ (Fig. 4).

Subsequent faulting is related to strike-slip movements along the NE-SW CF trend during the early Tortonian (10.04 ± 0.26 Ma), which corroborates published (though only relative) geochronological constraints^28,30,39^. Fault gouges associated with this phase (RGAK-Ar5) contain short-ordered illite-smectite with an illite content of 80%, indicating deformation at shallower crustal levels or colder fluids circulating within the faulted rock volumes at temperatures between 110 and 140 °C (Fig. 4).

The youngest dated faulting event reactivated inherited Chattian, E-W-trending BSFs during Late Zanclean-to-Chibanian shortening (between 3.68 ± 0.72 and 0.48 ± 0.77 Ma). During this phase, deformation took place at 70–90 °C, as indicated by the occurrence of random-ordered illite-smectite with an illite content of 30%^38,40^ (Fig. 4) in the dated fault gouges (RGAK-Ar1). Only the youngest dated gouge RGAK-Ar2 documents a higher deformation temperature (110–140 °C), since it contains short-ordered mixed layers illite-smectite with 80% illite (Fig. 4). This out-of-trend temperature likely suggests the involvement of high-T fluids at the time of the last recorded activation of the CF or the even later juxtaposition of a BSF formed at deeper structural levels.

K-Ar dating, illite polytype determination and composition and stacking order of mixed layers illite-smectite of fault gouges in adjacent BSFs allowed us to portray a time vs. temperature evolutionary history for the CF that documents a > 20 Myr-long polyphase faulting activity occurring at progressively colder, and presumably shallower, conditions (Fig. 4).

## Discussion

**Fig. 5:**
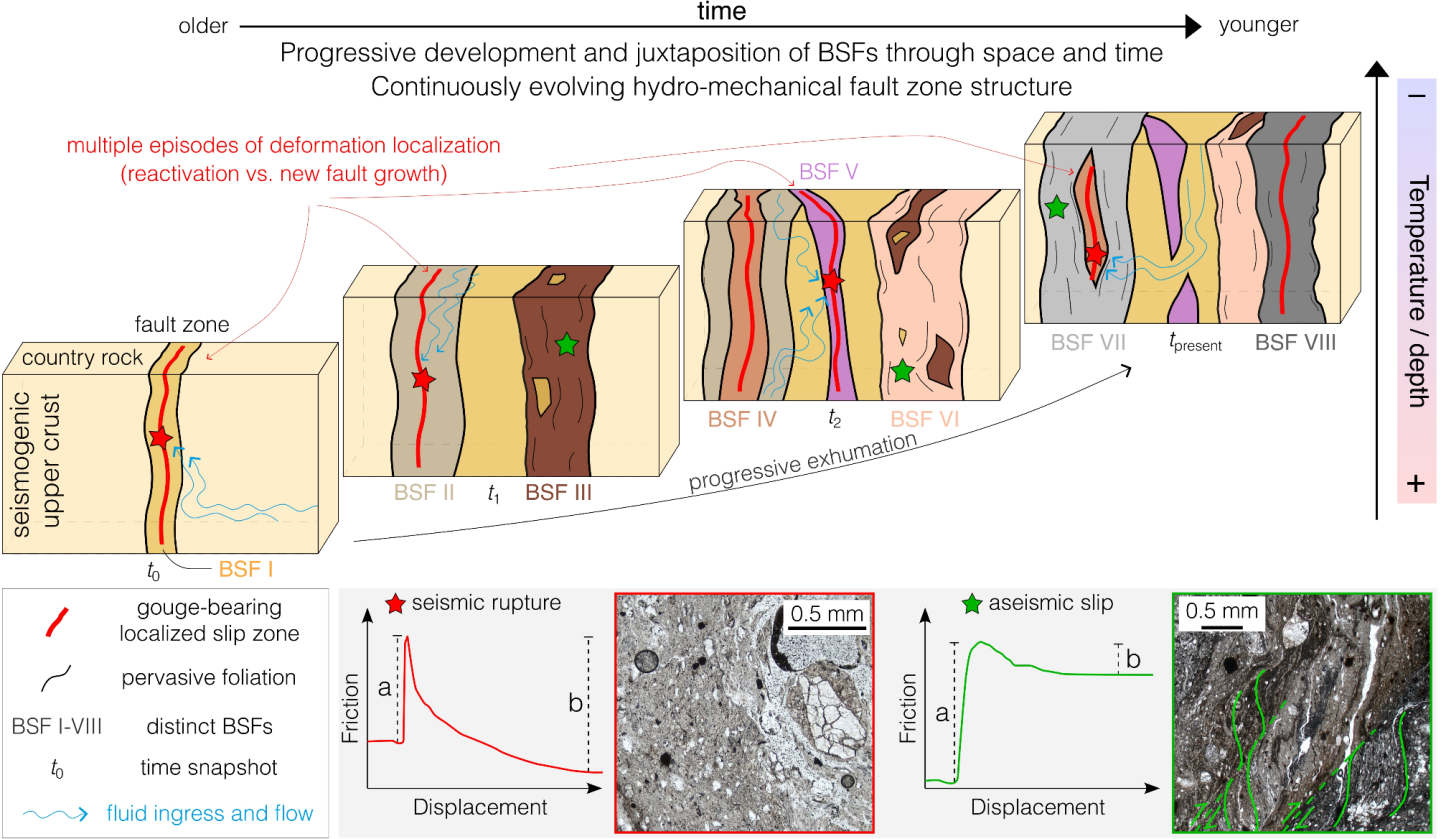
Conceptual model of fault zone evolution. According to this evolutionary scheme, the present-day exposure of tightly juxtaposed BSFs (labelled I through VIII with different colors) in an architecturally heterogenous fault (i.e., last snapshot - *t*_present_), represents the end-result of a protracted, cumulative and complex history encompassing multiple episodes of deformation localization and fluid(s) inflow and governing the possible occurrence of a spectrum of slip behaviors (insets): strong and competent BSFs promote a velocity-weakening frictional behavior (*a-b* < 0), which is a prerequisite for unstable, stick–slip behavior and earthquake nucleation, and exhibit localized deformation along discrete faults bearing gouge/cataclasite; weak and phyllosilicate-rich BSFs, on the other hand, promote a velocity-strengthening frictional behavior (*a-b* > 0) that may result in stable sliding and aseismic slip, and exhibit evidence of continuous and distributed deformation accommodated by foliated fabrics and frictional sliding (sketch of frictional behavior adapted from Collettini et al.^41^).

The most basic, yet central, message of this study is that, in mature faults, “juxtaposed” is not necessarily equivalent to “coeval” or even “genetically related”. Mature and long-lived fault zones are dynamic structural features embedding a complex patchwork of heterogeneous, non-coeval BSFs juxtaposed during multiple faulting episodes (Fig. 5), resulting in an architectural complexity that appears to be the norm rather than the exception, as documented by an increasing number of studies^1,8,11,41–44^. In-depth multiscalar and multi-technique studies are thus necessary to resolve this aspect. Ignoring or downplaying the time dimension of faulting may limit our understanding of the progressive evolution of faults in terms of mechanical and seismological perspectives and may possibly lead to inaccurate reconstructions of tectonic histories. The long-lasting and intricate evolution of the CF would have, in fact, remained undetected if it were not for a detailed characterization of its BSFs patchwork, which is vital before any sampling strategy for dating can be considered. We find that, despite multiple slip events and reactivation, local fault compartments (e.g., gouge pockets) can be preserved within BSFs, thus allowing slip dating over the life span of faults (see also refs.^11,23,44^). Each BSF invariably represents a unique snapshot of the fault’s long-lived history, revealing changes in deformation style (coaxial vs. non-coaxial), regime (semi-brittle vs. brittle) and distribution (anastomosed foliated rocks vs. discrete slip surface) through time and across the fault zone width. The lesson learned from studying cross-cutting BSFs within the CF demonstrates our ability to discern inherited compositional, textural and geochemical assemblages within reactivated faults, thereby enhancing our understanding of fault structure and heterogeneity.

This study provides a methodological benchmark for future investigations dealing with both active and fossil faults around the globe with implications reaching well beyond academia.

The high-resolution, time- and temperature constrained characterization of complex fault architectures makes it possible to (i) gain insights into the spatiotemporal variations of fault mechanical and fluid flow properties associated with evolving complex fault architectures and (ii) derive original and possibly unexpected constraints upon the hydro-mechanical behavior of rupturing during deformation localization in the brittle seismogenic crust.

During progressive deformation (Fig. 5) BSFs can develop gradually and become juxtaposed against one another. This can lead to fault zones with heterogenous (i) structural grain, (ii) mineral assemblage (Fig. 3a and Supplementary Figs. 1 and 2) and resulting diverse mechanical and frictional properties (Fig. 5; e.g., refs.^41,45^), (iii) fault rocks (in Fig. 5, compare gouge-bearing localized slip zone in quartz-rich BSFs with pervasive foliation development in clay-rich BSFs; see also Supplementary Fig. 4; e.g., ref.^46^) and (iv) permeability [Fig. 2f; e.g., parallel-to-foliation fluid flow would be promoted when lithons are embedded within foliation planes or hindered when lithons are (nearly) undeformed; e.g., refs.^8,47,48^].

Such heterogenous fault architectures may (i) result in complex 3D permeability structures variably impacting on fault zone hydraulic compartmentalization (with the development of rock domains that are more prone to fluid ingress and flow vs. low-permeability barriers where overpressure may build up)^8,47,48^ and (ii) generate local stress perturbations and steer fault slip behavior^42,45,49^, promote slow earthquakes and govern their spatial and temporal propagation^17,50^, control the propagation of afterslip^4^and seismic swarms^6^, produce fault weakness associated with reduced fault stability^1,5^and induce the occurrence of earthquake ruptures vs. slow-slip transients or aseismic slip^41^. The interplay between fluids and heterogeneous faults can, in turn, lead to a broad spectrum of complex and diverse slip behaviors^6,18–20^.

More importantly, petrophysical and rheological heterogeneities and anisotropies between and within BSFs suggest that the hydraulic and slip behavior of a fault may continuously evolve both in space (e.g., depending on the lateral continuity and interdigitation of BSFs and their internal compositional and structural heterogeneity) and during the entire fault life span (e.g., depending on fault rejuvenation due to the development and juxtaposition of younger BSFs; Fig. 5).

On this matter, some authors^4–6^ stressed that fault slip behavior and the dynamic evolution of earthquake swarms and aftershocks remain largely enigmatic to us because of our inadequate understanding of fault architecture. Numerical simulations^18,51,52^ also highlight the need of considering more realistic fault structures when dealing with fluid-induced seismic and aseismic fault movements. Those studies, however, basically focus on seismological or experimental data and tend to neglect the complex space and time dimension of faulting by considering rather static snapshots of a long-lasting, cumulative evolution.

Our approach is thus of great utility, it is timely and is capable to assist studies aiming at better characterizing the 4D perspective on rheology, fluid flow and seismotectonic patterns in the brittle crust (with clearcut implications upon seismic hazard assessment), as well as the thermal conditions during faulting and related exhumation histories. In particular, it allows to build of more realistic and robust conceptual and physical fault models to be (1) implemented when sampling for- and interpreting laboratory friction experiments aimed at characterizing their rheology^1,41,49^ and (2) incorporated into simulations aimed at predicting their hydro-mechanical behavior, either in natural processes (e.g., earthquake cycle at shallow conditions or subduction zones, propagation dynamics of slow earthquakes) or industrial applications (e.g., H_2_/CO_2_storage, fault sealing, reservoir stimulation during unconventional geothermal energy and hydrocarbon production)^18,19,32,50,53^.

To conclude, the present-day exposure of complex fault architectures represents the end-result of a protracted, cumulative and complex history encompassing multiple episodes of deformation localization and fluid(s) inflow and governing the possible occurrence of a spectrum of slip behaviors (Fig. 5). Structural, mineralogical, thermal, petrophysical and mechanical data thus ought to be integrated over the lifespan of the studied fault system to generate a comprehensive evolutionary scenario.

## Methods

### Fault zone characterization

Our methodological approach builds upon the concept of Brittle Structural Facies (BSF)^11^, which describes tightly juxtaposed volumes of deformed rock characterized by a given fault rock type, composition, texture, color and age of formation. BSF is proven to be a powerful tool to unravel complex deformation histories archived in crustal brittle fault zones^8,11,43,44^. Detailed high-resolution field structural analysis of outcrop exposures of the CF was performed to identify and characterize different BSFs. At the outcrop, structural elements (e.g., faults, gouge lenses, foliation, folds) were characterized according to their type, attitude, associated deformation style, dimension and cross-cutting relationships. Aiming at better defining the heterogeneity of the BSFs and their fault rocks, thus, to derive more insights in the bulk and local hydro-mechanical behavior of complex fault architectures, we measured the in situ outcrop permeability. It was measured on BSFs, both parallel and perpendicular to the tectonic foliation and/or bedding (when preserved). This allowed us to explore the effect of planar anisotropies upon the permeability properties within the fault architecture. Our results only offer a first-order constraint on the present-day permeability at surficial conditions and, therefore, cannot be directly extrapolated to depth and back in time. Here, we use our data to grasp the magnitude order of permeability contrast among different BSFs within the fault zone. Further instrumental and operational details can be found in Supplementary Method 1; see also refs.^8,54^). Variably consolidated samples of BSFs and fault gouges therein were collected for microanalytical investigation (see below). Epoxy-impregnated thin sections (~ 30 μm thick) were cut parallel to the slip direction and perpendicular to the planar fabric, from oriented samples.

### Characterization of BSFs and fault gouges

The microstructure and mineralogical composition of BSFs and fault gouges were characterized by thin section and X-ray diffraction (XRD) analyses. Imaging was performed by means of optical microscopy (Nikon ECLIPSE C*i*POL) and scanning electron microscopy (Thermo Scientific™ Quattro S ESEM, hosted at the Dept. of Biological, Geological and Environmental Sciences of the University of Bologna). Particle sizes of < 0.1, 0.1–0.4, 0.4–2, 2–6 and 6–10 μm were analysed by XRD as randomly oriented samples, using a Bruker D8 Advance system (Cukα), housed in the X-ray laboratory of the Department of Earth Sciences in Sapienza University of Rome. Samples were X-rayed from 2 to 70 °2θ with a step size of 0.02 °2θ and a count time of 1 s per step at 40 kV and 30 mA. The X-ray quantitative analysis of the grain size fractions and illite polytype determination were performed by Profex-BGMN Rietveld refinement software^55^. Profex is a graphical user interface for quantification of powder XRD data, that calculates the weight% of minerals. The software fits the sum of stored XRD patterns of standards (calculated pattern) to the measured tracing by varying the contribution of each standard pattern. Profex allows to select different default structure files (minerals) available in the BGMN database, described by different freely editable parameters (particles size and orientation, micro-strain, peaks sharpness, etc.; more details in Supplementary Method 2 and Supplementary Fig. 8).

### Conversion to paleotemperatures

Clay minerals are mainly sensitive to temperature, and the use of mixed layers illite-smectite and the transformation sequence dismectite - random - ordered mixed layers illite-smectite (R0) - ordered mixed layers illite-smectite (R1 and R3) - illite-di-octahedral K-mica (muscovite) as indicator of maximum paleotemperature condition is widely accepted^55–58^. In fact, changes in the composition of mixed layering, layer expandability, and illite-smectite ordering are empirically related to temperature changes due to burial, shear heating or hydrothermal fluids^58,59^. Random ordered illite-smectite (R0) with illite content lower than 50% indicates early diagenetic conditions at temperature lower than 100 °C, short-range ordered illite-smectite (R1) indicates deep diagenetic conditions at temperature between 100 and 110 °C and 170–180 °C, long-range ordered (R3) illite-smectite forms at temperature higher than 170 °C during progressive burial^38,40,59^ or at lower temperature (> 140–150 °C) during short-lived heating events as in areas of relatively recent thermal activity and elevated geothermal gradients and/or hydrothermal environments^58^. Discrete illite indicate temperature > 210 °C and muscovite > 275°C^38^.

### Fault gouge K-Ar dating

Fault rocks were disaggregated by repeated freeze–thaw cycles, and clays were later suspended in deionized water. Sedimentation with Stokes law and a combination of continuous flow and fixed-angle rotor centrifuging were used to produce particle sizes of < 0.1, 0.1–0.4, 0.4–2, 2–6 and 6–10 μm. Argon extraction was carried out from clay aliquots packed in Mo foil within a stainless steel ultra-high vacuum line using a Pond Engineering furnace at 1400 °C. The sample gas was first purified using a titanium sublimation pump and later with one SAES GP50 getter at room temperature and one SAES GP50 getter at 350 °C. Sample gas was spiked with approximately 2 × 10^−13^ moles of pure ^38^Ar (ref.^60^). and analyzed in an IsotopX NGX multicollector noble gas mass spectrometer fitted with five Faraday cups for 600 integrations of 1 s each. Nominal Ar beam intensities were determined using a second-degree polynomial regression to gas inlet time zero with an in-house Python program (Geological Survey of Norway). Beam intensities for ^38^Ar and ^36^Ar were corrected for mass discrimination relative to ^40^Ar by a power law^61^, using the weighted mean ^40^Ar/^36^Ar ratios of 299.781 ± 0.014 measured from atmospheric Ar in an online air pipette and the atmospheric Ar composition of Lee et al.^62^. Radiogenic ^40^Ar^∗^concentrations and their uncertainties were calculated using the equations outlined in Hałas and Wójtowicz^63^. Within this analytical batch, four aliquots of GA-1550 biotite (98.5 ± 0.5 Ma)^64^ were analyzed and yielded a weighted mean age of 98.53 ± 0.36 Ma. Potassium (K) concentrations were determined by fusing an aliquot of approximately 50 mg in Lithium tetraborate at ∼ 1000 °C to form a glass, which was then dissolved in HNO_3_with a Rhodium internal standard at 5 ppm prior to analysis with an Agilent 5110 VDV inductively coupled plasma optical emission spectrometer (ICP-OES). K–Ar dates were calculated using the ^40^K abundance and decay constants of Steiger et al.^65^. More detailed analytical procedures for K-Ar dating can be found in Viola et al.^66^.

### Illite age analysis

The effects of potential host rock contamination, i.e., mixing between authigenic and detrital illite and muscovite inherited from the host rock, may be resolved through the Illite Age Analysis (IAA) approach^13,25,67,68^. The IAA discriminates between the mostly detrital 2M_1_ polytype (which in turn might represent a mixture of authigenic high-temperature illite and cataclastic, synkinematic muscovite) and a truly authigenic phase 1M_d_ formed during brittle faulting (Supplementary Fig. 5). To estimate the age and uncertainty of synkinematic illite‐1M_d_ and detrital illite‐2M_1_, we normalized to 100% the proportion of illite‐2M_1_ and illite‐1M_d_ determined by XRD analysis plotting the data as apparent K‐Ar date versus percent of detrital illite, and linearly extrapolated to 0% and 100% illite‐2M_1_by the York et al.’s^69^ regression method. The latter accounts for the uncertainties on the individual data points and assumes a Gaussian distribution of the uncertainty envelopes in both x and y coordinates which are valid for the quantified XRD mineral proportions.

## Electronic supplementary material

Below is the link to the electronic supplementary material.

Supplementary Material 1

## Data Availability

All data are included in the main text and in the Supplementary Information and stored in the figshare public repository 10.6084/m9.figshare.27055180.v3.
