# Supplementary material for: High-resolution investigations of fault architecture in space and time
Source: Sci Rep. 2025 Jan 17;15:2258. doi: 10.1038/s41598-025-86104-w (PMC11748627; doi:10.1038/s41598-025-86104-w)
Supplement: Supplementary file 1 — Supplementary Material 1 [file 41598_2025_86104_MOESM1_ESM.pdf]

Supplementary Information for

High-Resolution Investigations of Fault Architecture in Space and Time

Leonardo Del Sole[1*], Giulio Viola[1*], Luca Aldega[2], Vincenzo Moretto[2], Manuel Curzi[2], Ruikai Xie[3], Luigi Cantelli[1], and Gianluca Vignaroli[1]

[1]Dipartimento di Scienze Biologiche, Geologiche ed Ambientali, Università di Bologna, Bologna, Italy. [2]Dipartimento di Scienze della Terra, Sapienza Università di Roma, Rome, Italy. [3]Geological Survey of Norway, Trondheim, Norway.

*e-mail: leonardo.delsole@unibo.it; giulio.viola3@unibo.it

This PDF file includes:

Supplementary Note 1

Supplementary Methods 1 and 2

Supplementary Data 1

Supplementary Figures 1 to 8

Supplementary Tables 1 to 3

Supplementary References

Supplementary Note 1. Geological framework

The Betic Cordillera in southern Spain is the northern branch of the Betic-Rif orogen, which forms the westernmost termination of the Alpine belt in the Mediterranean (Fig. 1). The Betics formed during c. N-S Africa-Eurasia convergence since the Late Cretaceous[1], and consist of two major domains, the Internal (Alborán Domain) and External zones (Fig. 1). The former is an orogenic wedge resulting from Eo-Oligocene[2] or Eocene-Early Miocene[3] subduction and crustal stacking of three major nappe complexes, which, from top to bottom, are the Maláguide, the Alpujárride and the Nevado-Filábride. The External Zone is a non-metamorphic, thin-skinned fold and thrust belt that accommodated early-middle Miocene continental collision-related shortening involving the westward-drifting Alborán Domain and the Iberian margin (Iberian Massif, Fig. 1)[4,5]. Since the Early Miocene, thrusting in the External Zone was concurrent with extension in the hinterland, in response to Ligurian-Tethys slab rollback[2,4] and lithospheric delamination[6]. Crustal extension was accompanied by calc-alkaline magmatism and led to the opening of the Alborán Sea Basin[7]. From the latest Miocene, NNW-SSE compression caused crustal thickening and regional uplift (Fig. 1)[4,8].

The CF is part of the Eastern Betic Shear Zone (Spain; Fig. 1), the Betic segment of the lithospheric, sinistral transpressive Trans-Alborán shear zone[9], which is a diffuse plate boundary system accommodating the still ongoing NNE-directed Gibraltar slab dragging by the African plate[10]. The Eastern Betic Shear Zone extends over 250 km from Almeria to Alicante and is characterized by the en-echelon arrangement of its constituent segments, which include the Carboneras, the Palomares, Alhama de Murcia, Carrascoy - Bajo Segura and Crevillente faults (Fig. 1). Currently available relative chronological constraints outline a sketchy (dominantly strike-slip) faulting history spanning the Early Miocene-present[11-13], which is characterized by both Middle Miocene dextral and late Tortonian to Recent sinistral movements[14]. In the study area, the CF deforms Paleozoic to Mesozoic basement rocks of the Alborán Domain nappe complexes (variably affected

by pre-Alpine and Alpine metamorphism)[15], Middle-to-Upper Miocene post-orogenic sedimentary successions and Burdigalian-to-Messinian volcanic rocks (Fig. 1)[11,12,16].

Supplementary Method 1. In situ permeability measurements

In situ air permeability was measured with a TinyPerm-3 portable handheld air permeameter (*New England Research, Inc.*) calibrated by the manufacturer with known standards. The instrument allows for the investigation of rock (bulk) permeability in the ~10^{-11}–10^{-15} m^2 (10^{-3}–10^{1} Darcy) range within volumes of rock of 1–1.5 cm^3, even though controlled laboratory tests have demonstrated its capability to measure values as low as ~10^{-17} m^2 (10^{-5} D)[17]. The air permeameter directly yields an estimate of the permeability based on the outgoing air flow rate from the built-in compression vessel. Measuring sites were systematically cleaned to avoid potential effects of superficial alteration that could result in spurious results. Permeability values obtained from air permeametry are not directly comparable with values measured by means of laboratory tests on rock plugs or image analysis without standardization[17,18]. Therefore, here we use our data to grasp the magnitude order of permeability contrast among different BSFs within the fault zone. Permeability results are illustrated in Fig. 2F and listed in Supplementary Table 1. Boxplots were made with the *R* software[19]. The black thick line is the 2nd quartile (median), the lower and upper box (interquartile range) sides are the 1st and 3rd quartiles, respectively. The lower and upper whiskers define, respectively, the minimum and maximum observed values in the data range (i.e., *c.* $\pm 2.7\sigma$ and 99.3% coverage if the data are normally distributed) excluding the outliers (white circles).

Supplementary Method 2. Details for Rietveld refinement of fault gouges by Profex

Profex is a graphical user interface for quantification of powder XRD data, that calculates the weight percent of minerals. The software fits the sum of stored XRD patterns of standards (calculated pattern) to the measured tracing by varying the contribution of each standard pattern. Profex allows to select different default structure files (minerals) available in the BGMN database, described by different freely editable parameters

(particles size and orientation, micro-strain, peaks sharpness, etc.). For mixed layers illite-smectite (I-S), no default structure files are available in the BGMN database, thus we referred to the illite-smectite structure files developed by Ufer et al. [20], who introduced different models from R0 to R3 I-S with intermediate starting values for the refinable parameters (Supplementary Fig. 8). For sample RGAK-Ar6 (Supplementary Fig. 6), we applied a bimodal refinement for different crystal sizes of the same mineral (muscovite-$2M_1$). This was due to: 1) decreasing K-Ar dates towards the finer fractions (from 117.4 Ma in the 6-10 μm grain-size fraction to 33.1 Ma in the <0.1 μm grain-size fraction) all younger than the rock formation age (Paleozoic) that indicates the occurrence of neoformed mineral phases in all the fractions, 2) bad Rietveld refinement by using one single muscovite-$2M_1$ phase, and by editing crystallite size (B1), micro-strain (k2) and preferred orientation (SPHAR) implying the coexistence of two muscovite-$2M_1$ with different crystallite size. To perform a bimodal refinement, we modified the structure file by inserting a command string pre-set by the program (Supplementary Fig. 6) as indicated by the Profex user manual in "advanced refinements and features". We interpreted the muscovite with the greater crystallite size as detrital, and that with smaller crystallite size as neoformed. The bimodal refinement provides a total weight percent for the two phases. To calculate the relative amount of detrital and neoformed muscovite-$2M_1$, we calculate peak areas of both mineral phases and applied the formula $(Wt.\%)/A = X/N$ (Supplementary Fig. 6) where the total content of detrital and neoformed muscovite-$2M_1$ provided by Rietveld refinement (Wt.%) is proportional to the total peak area (A) of detrital muscovite-$2M_1$ and neoformed muscovite-$2M_1$ (N).

Supplementary Data 1. Details of fault gouge mineral assemblage

XRD data are shown in Supplementary Fig. 2 and listed in Supplementary Table 2.

RGAK-Ar1 and RGAK-Ar2 contain muscovite-$2M_1$ (25-61%), quartz (21-47%), dolomite (2-5%), hematite (1-7%), rutile (1-2%), paragonite (2-6%), chlorite (2-8%) and kaolinite (1-8%) in the 6-10 μm and 2-6 μm grain-size fractions that progressively decrease their content or disappear in the 0.1-0.4 μm and <0.1 μm fractions

(Supplementary Fig. 2). The synkinematic mixed layers illite-smectite (R0 I-S with an illite content of 30% for RGAK-Ar1 and R1 I-S with an illite content of 80% for RGAK-Ar2) occur in the finer sub-fractions. The finest grain-size fraction consists of R0 illite-smectite (50%), muscovite-$2M_1$ (37%) and chlorite (13%) for RGAK-Ar1 and is made up of R1 illite-smectite (67%), muscovite-$2M_1$ (28%) and chlorite (5%) for RGAK-Ar2 (Supplementary Fig. 2). RGAK-Ar6 contains a mineral assemblage composed of quartz, hematite, rutile, chlorite and inherited and neoformed muscovite-$2M_1$ (Supplementary Fig. 6). Quartz, chlorite, detrital muscovite-$2M_1$ and hematite become progressively less abundant in the finer fractions (0.4-2, 0.1–0.4 and <0.1 µm) where a general increase of neoformed muscovite-$2M_1$ is instead observed from 61% to 89%. RGAK-Ar4 is mostly made up of quartz (44-56%), muscovite-$2M_1$ (23-35%) and chlorite (9-11%) and subordinate amounts of ankerite, hematite, rutile, pyrite, paragonite and kaolinite not exceeding 4% in the coarser 6-10 µm and 2-6 µm fractions. Synkinematic minerals are represented by R0 illite-smectite with an illite content of 30% that is observed from the 0.4-2 µm to the finest fraction. RGAK-Ar5 contains muscovite-$2M_1$ (44-51%), quartz (26-34%), chlorite (16-17%), albite (3%), hematite (0-1%), paragonite (1-2%) and rutile (1%) in the 6-10 µm and 2-6 µm grain-size fractions. Synkinematic minerals are R1 illite-smectite with an illite content of 80% that progressively increase their amount to 60% from the 0.4-2 µm to the <0.1 µm fraction, whereas non-clay minerals such as quartz, albite, hematite, rutile and paragonite disappear from the 0.1-0.4 µm fraction.

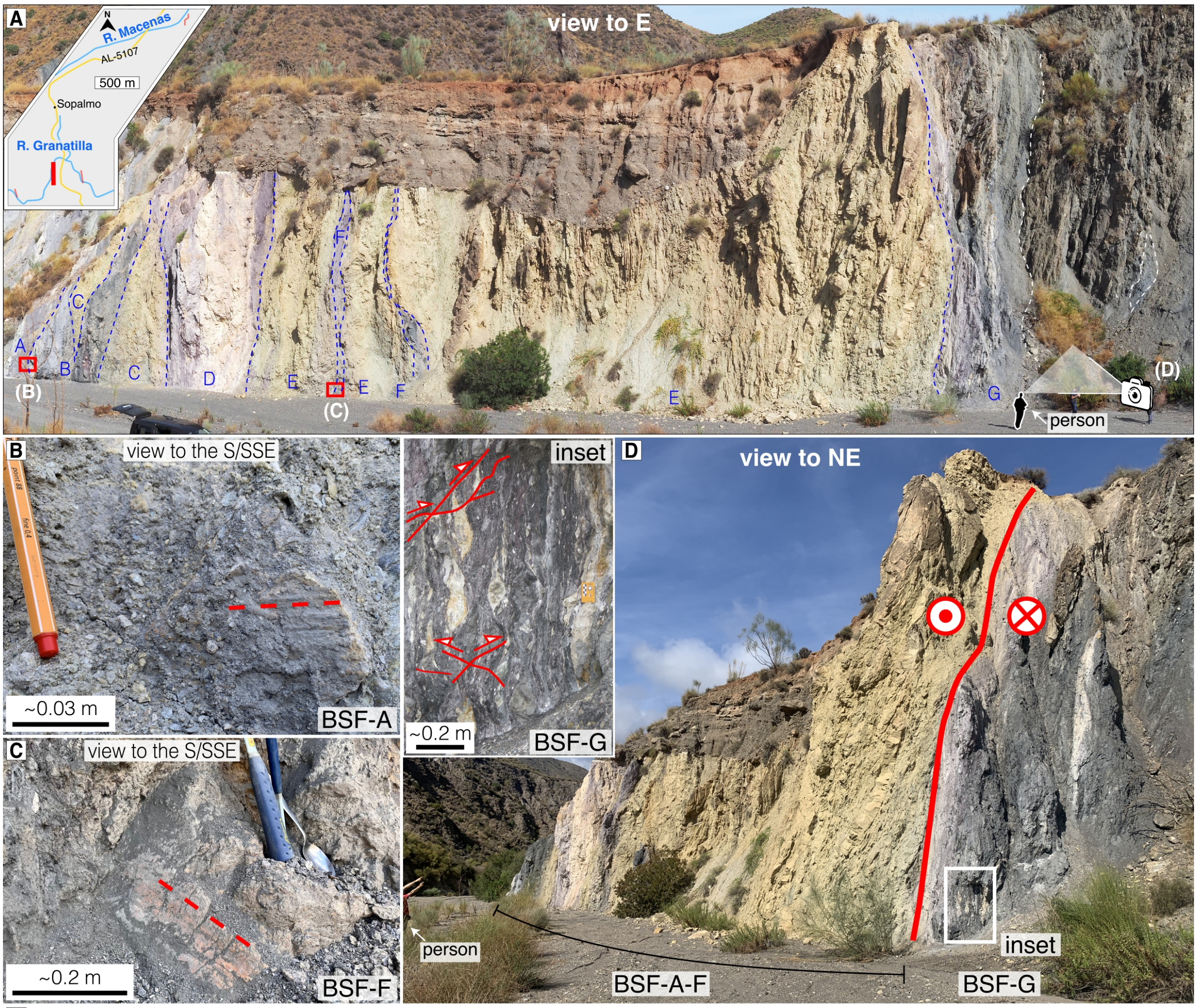

E

BSF	Lithotype
A	phyllite
B	phyllite and sedimentary rocks (clasts)
C	sandstone, siltstone, mudstone, marl and volcanoclasitc rocks
D	phyllite, quartzite
E	siltstone, marl, sandstone, conglomerate
F	phyllite
G (and localized gouge S of BSF-G)	phyllite, schist

Supplementary Figure 1. Outcrop RGA. (A) Uninterpreted outcrop. (B and C) Faint evidence of strike-slip kinematics (abrasion striae with pitch > 170° or < 20°) is observed along the E-W to ENE-WSW foliation planes in BSFs A through to F. (D) BSF-G is characterized by a NE-SW-striking ("N040°") steeply dipping foliation that cuts across the E-W trending fabric. NE-SW striking, relatively late and low-angle reverse faults (commonly in conjugate pairs) crosscut both the E-W and NE-SW high-angle foliation planes. (E) Main rock types of each BSF.

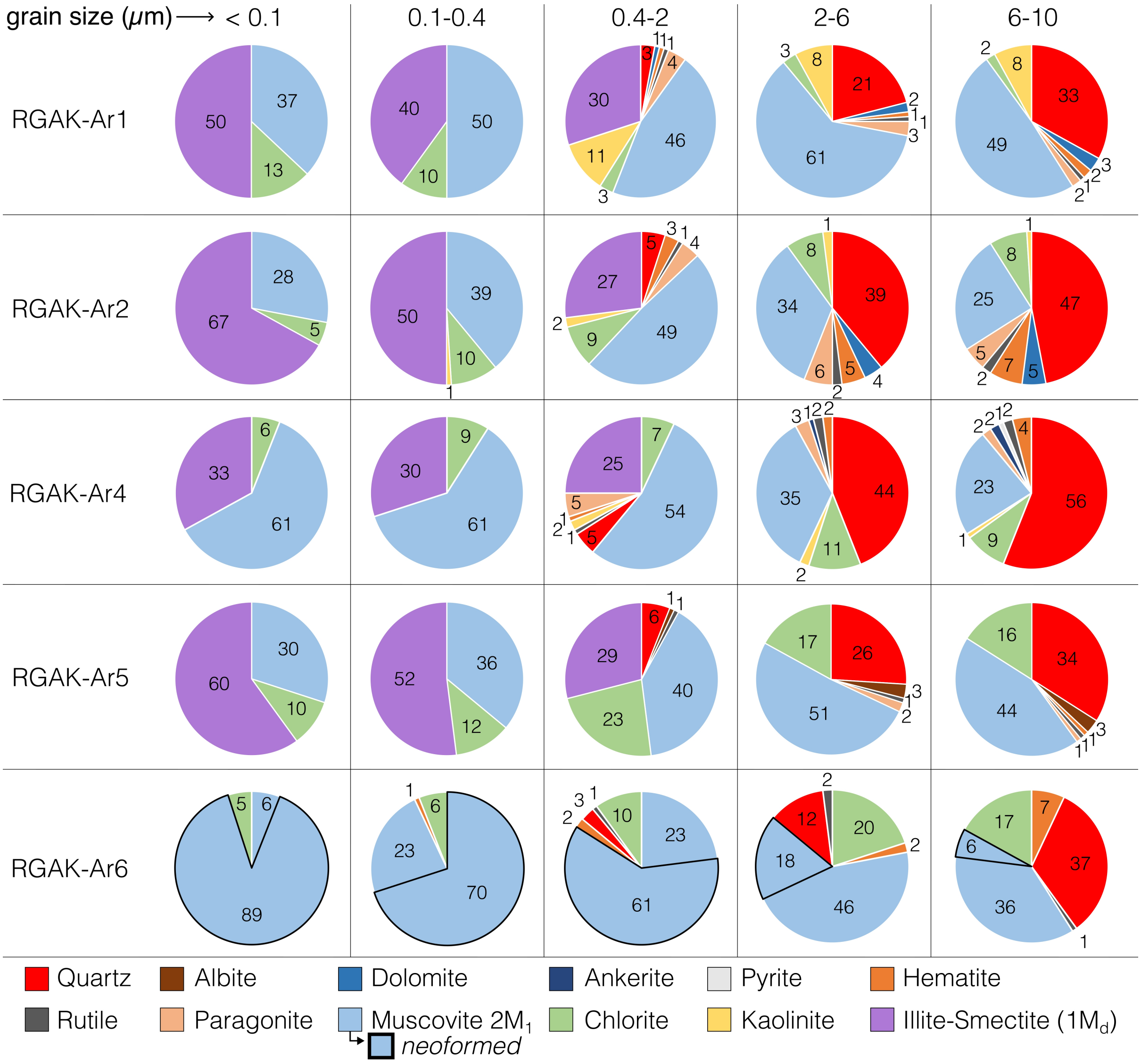

Supplementary Figure 2. XRD quantitative analysis (%wt) of the dated grain size fractions of each gouge sample and illite polytype determination.

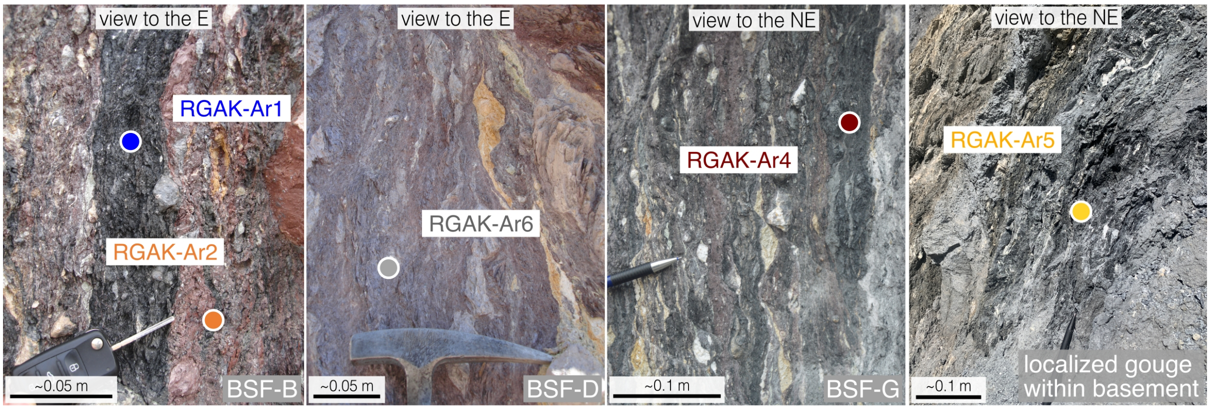

Supplementary Figure 3. Outcrop setting of the dated gouge samples. Close-up on the sampling sites of the dated gouge samples (circles labelled as in Fig. 3).

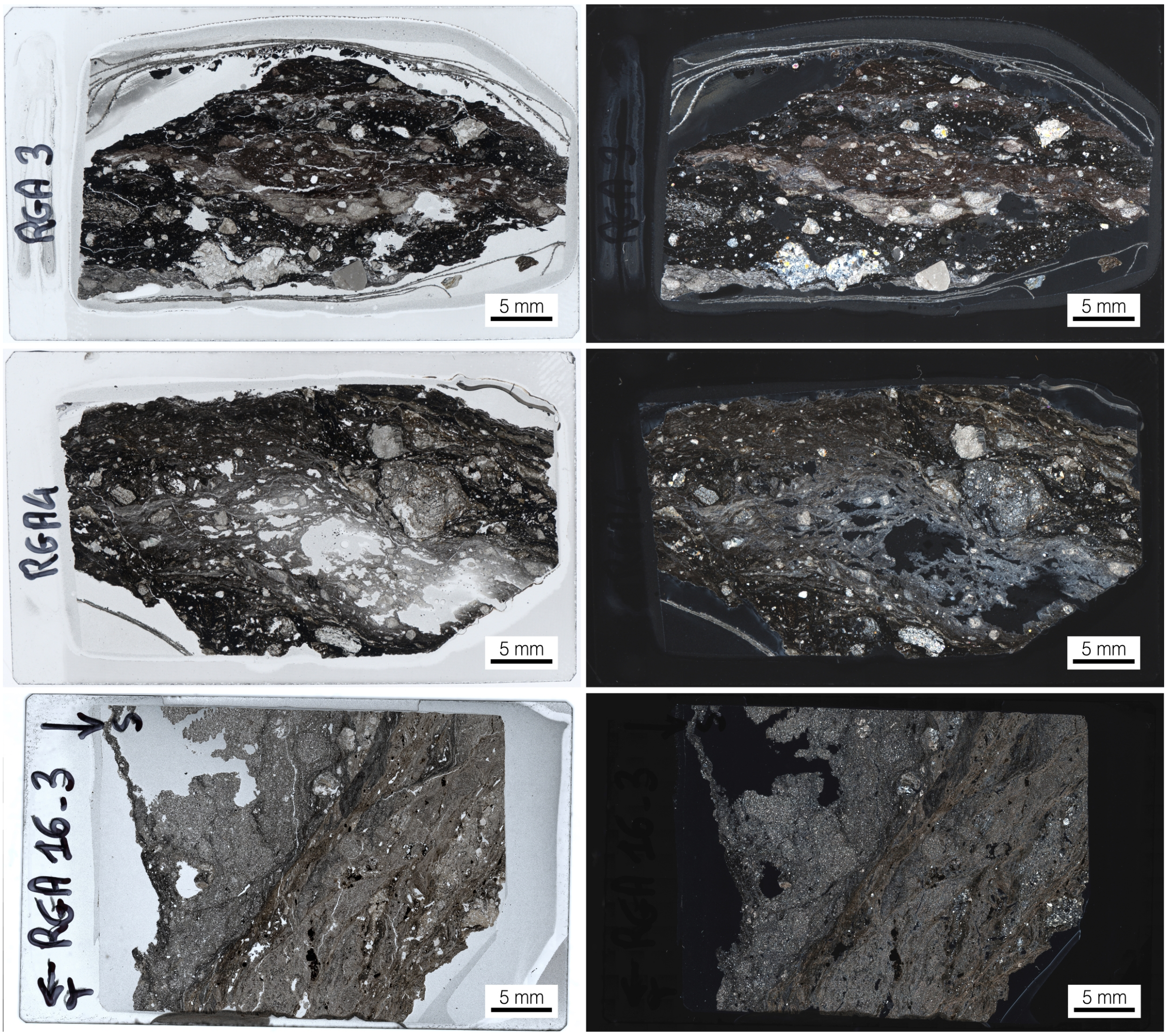

Supplementary Figure 4. Representative fault gouge microphotographs. Whole-section mosaics assembled by optical microscopy in plane polarized light (left) and crossed polarized light (right).

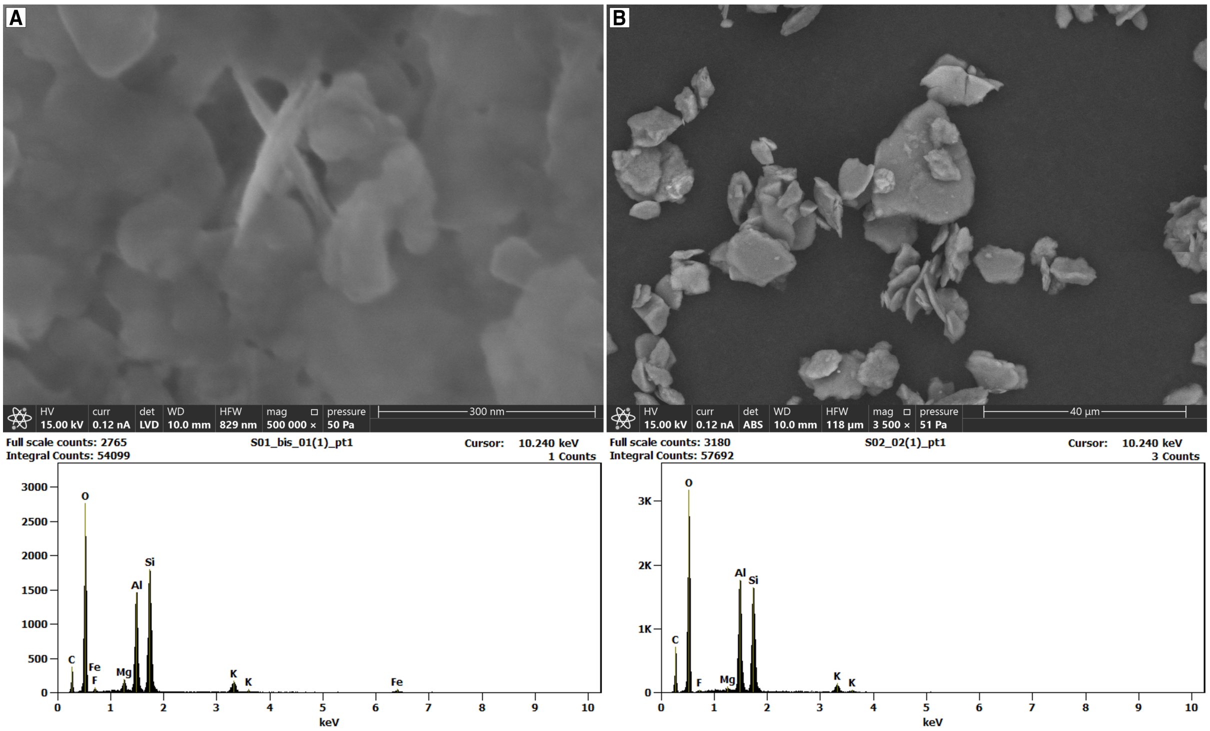

Supplementary Figure 5. Representative SEM microphotographs of authigenic/synkinematic illite (A) and detrital muscovite (B) with associated EDS spectra for, respectively, the <0.1 µm and 6-10 µm fractions of dated gouge samples. Illite-smectite ($1M_d$) occurs with predominantly prismatic platy habit suggesting in situ neo-crystallization[21,22], while muscovite ($2M_1$) tends to have more equant habit.

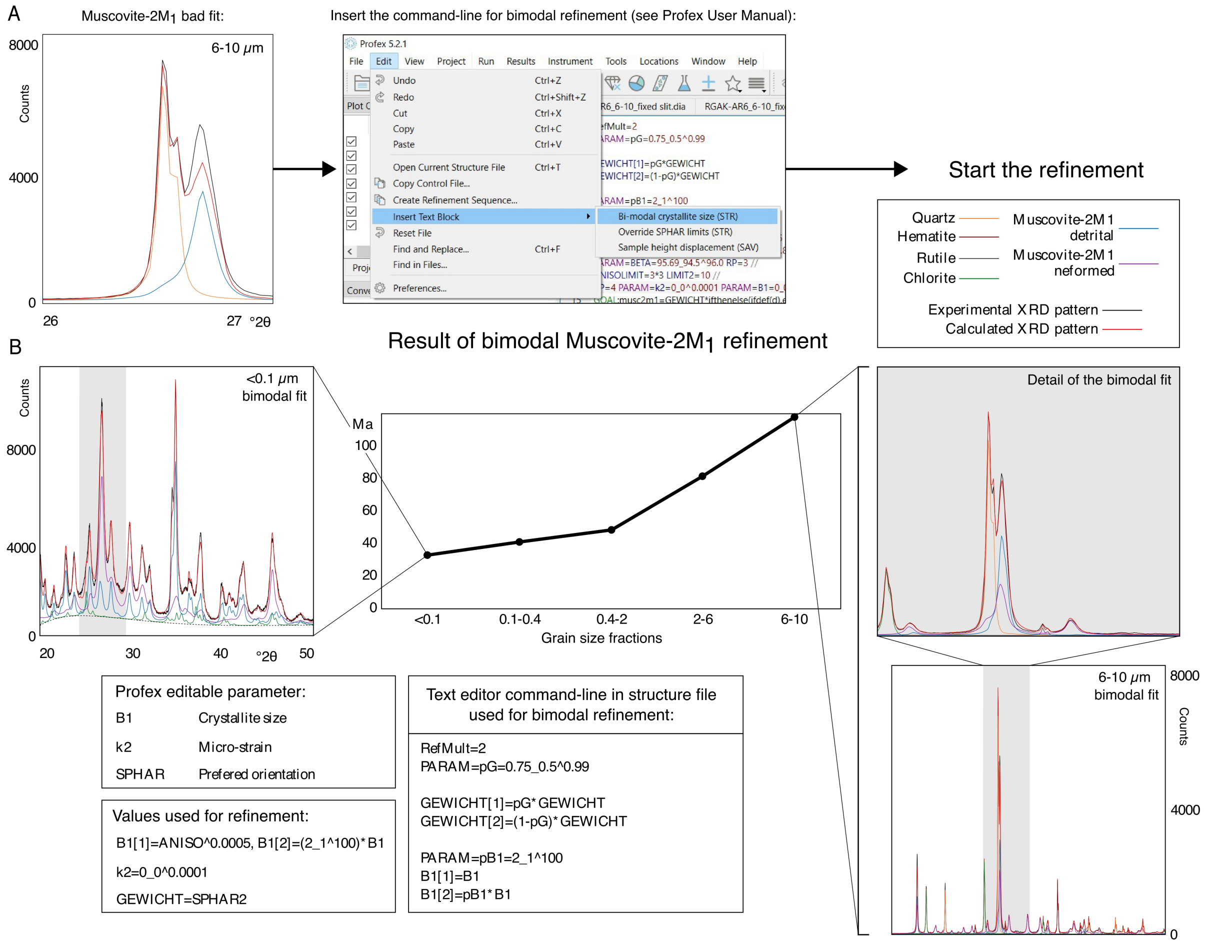

Supplementary Figure 6. (A) Work-flow for bimodal Rietveld refinement of X-ray peaks (in this case muscovite-2M_1 in the 26-27° 2θ interval), as described in the Profex User Manual[23]. (B) Bimodal refinement of sample RGAK-Ar6 by using a neoformed and detrital muscovite-2M_1 with different crystallite size, micro-strain and orientation. Editable parameters in the BGMN structure file in Profex and values used for bimodal refinement are shown in the boxes.

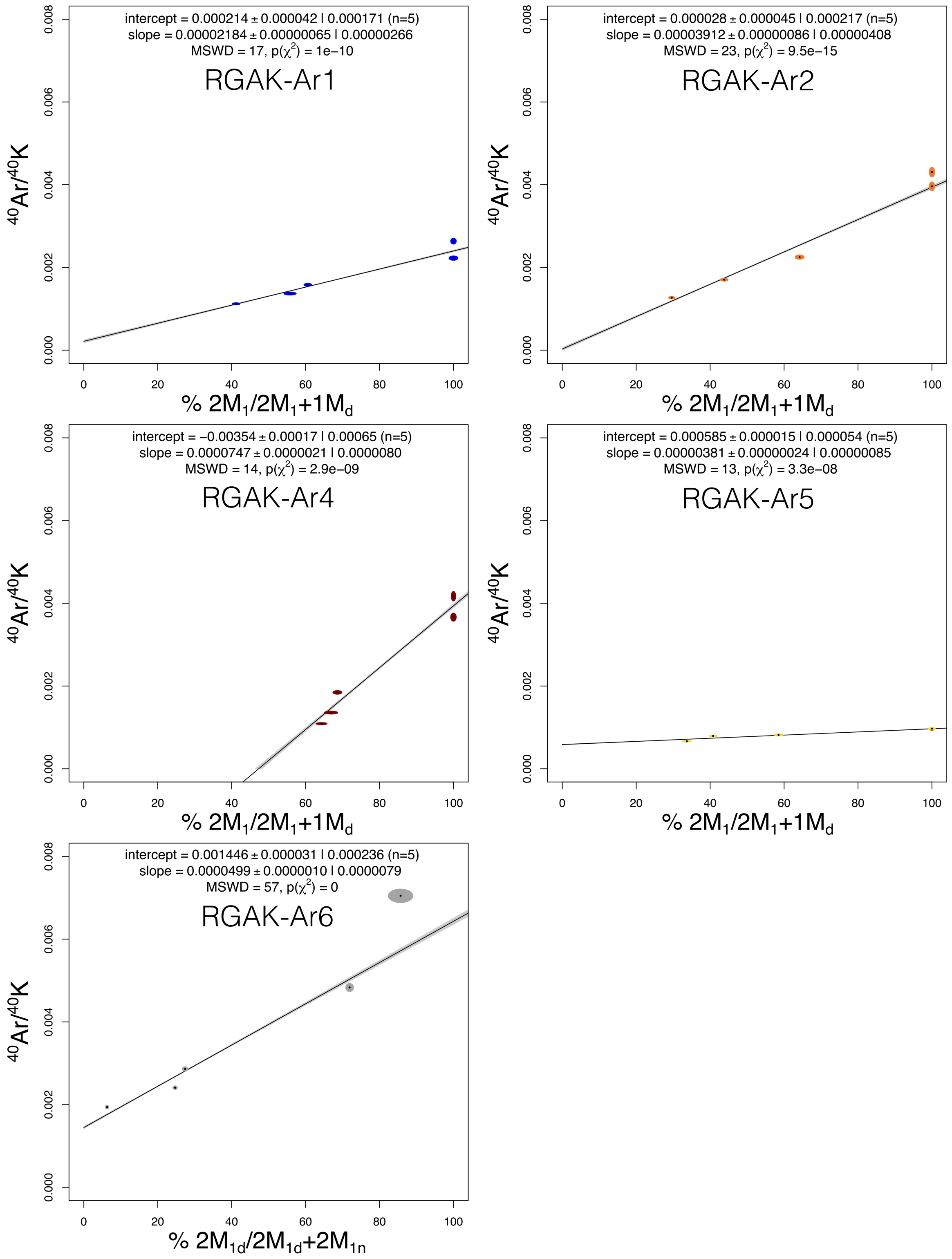

Supplementary Figure 7. Illite Age Analysis (IAA) data. $^{40}Ar/^{40}K$ ratios are plotted against percentage of illite-$2M_1$ (normalized to 100%). For sample RGAK-Ar6, $^{40}Ar/^{40}K$ ratios are plotted against percentage of muscovite-$2M_1$ (normalized to 100%). See the main text for details. Plots made with IsoplotR v.5.6[24].

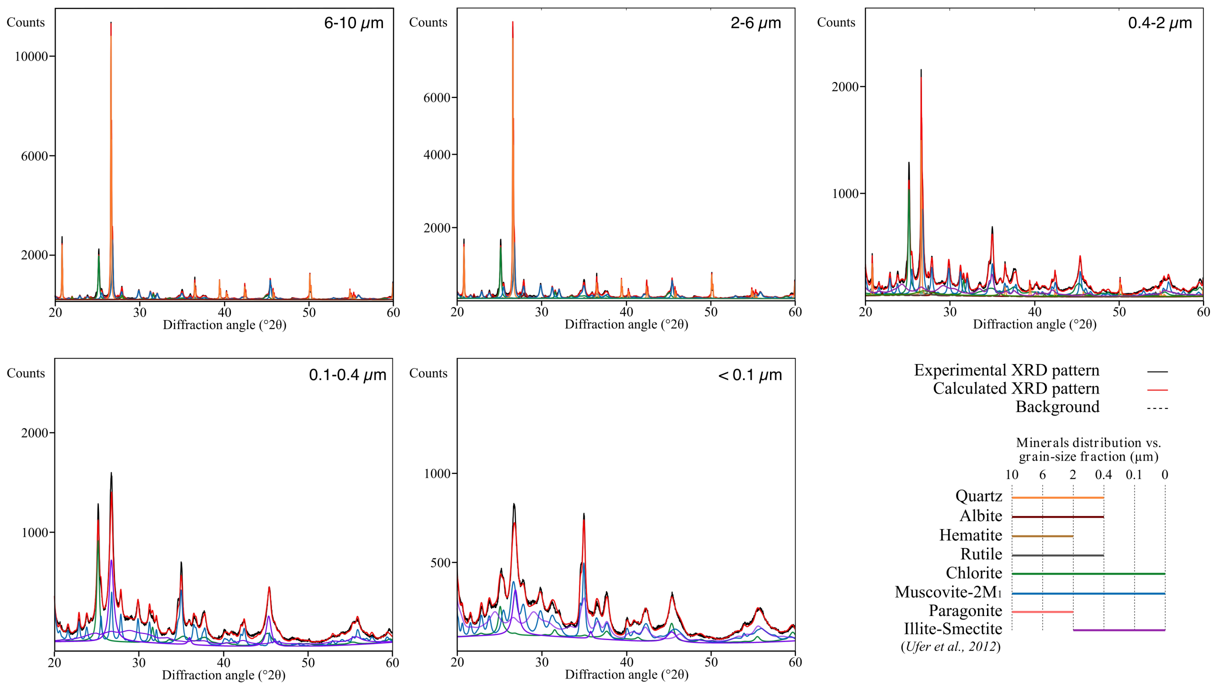

Supplementary Figure 8. Rietveld refinement for sample RGAK-Ar5, showing the decreasing content of detrital minerals from the 6-10 µm to the <0.1 µm grain-size fraction (quartz, albite, pyrite, etc.), and the occurrence of neoformed illite-smectite with illite-1M_d polytype for the 0.4-2, 0.1-0.4 and <0.1 µm grain-size fractions.

Supplementary Table 1. Summary of *in situ* permeability measurements at outcrop RGA.

BSF	Structural element	Permeability (m^2)	Permeability (D)
B	Measurements parallel to the foliation (balck gouge band; Fig. 2B)	4.01E-12	4.06E+00
		6.37E-12	6.45E+00
		4.24E-11	4.30E+01
		3.67E-11	3.72E+01
		1.44E-12	1.46E+00
		2.24E-12	2.27E+00
		1.16E-12	1.18E+00
		7.94E-13	8.04E-01
		3.00E-12	3.04E+00
		2.45E-12	2.48E+00
		2.18E-14	2.21E-02
		7.96E-13	8.07E-01
	Measurements parallel to the foliation (reddish gouge band; Fig. 2B)	4.01E-13	4.06E-01
		4.34E-13	4.39E-01
		1.65E-13	1.67E-01
		3.23E-13	3.27E-01
		6.13E-13	6.21E-01
		6.09E-13	6.17E-01
		4.92E-13	4.98E-01
		2.26E-13	2.29E-01
		4.38E-13	4.43E-01
		6.52E-13	6.61E-01
	Clasts embedded in the foliated matrix (Fig. 2B)	1.64E-14	1.67E-02
		1.61E-14	1.63E-02
		1.09E-14	1.11E-02
		2.06E-13	2.08E-01
		1.69E-14	1.71E-02
		1.01E-13	1.02E-01
		1.33E-13	1.34E-01
		2.07E-14	2.10E-02
		1.31E-12	1.33E+00
		1.22E-12	1.23E+00
		4.50E-13	4.56E-01
C	Bulk (massive sandstone-to-mudstone, marl, volcanoclastic rocks)	2.05E-13	2.08E-01
		2.28E-13	2.31E-01
		3.39E-14	3.43E-02
		1.41E-12	1.43E+00
D	Measurements parallel to the foliation (greenish gouge; Fig. 2C)	7.60E-13	7.70E-01
		1.08E-12	1.09E+00
		1.19E-12	1.20E+00
		4.27E-13	4.33E-01
		1.44E-12	1.46E+00
		7.36E-13	7.46E-01

		3.44E-13	3.49E-01
		5.94E-13	6.02E-01
		4.14E-13	4.19E-01
		7.72E-13	7.82E-01
	Measurements parallel to the foliation (violet gouge; Fig. 2C)	8.52E-13	8.64E-01
		1.73E-13	1.75E-01
		1.21E-13	1.22E-01
		1.89E-12	1.92E+00
		4.85E-13	4.91E-01
		3.39E-13	3.43E-01
		5.54E-13	5.62E-01
		4.42E-13	4.48E-01
		7.01E-13	7.10E-01
		3.00E-13	3.04E-01
	Measurements orthogonal to the foliation (violet gouge; Fig. 2C)	3.63E-13	3.68E-01
		3.81E-13	3.86E-01
		1.41E-13	1.43E-01
	Bulk (massive, white pulverized domain; Fig. 2C)	3.47E-13	3.51E-01
		1.88E-13	1.90E-01
		2.25E-12	2.28E+00
		6.44E-13	6.52E-01
		3.90E-13	3.95E-01
		1.10E-12	1.11E+00
		1.33E-12	1.35E+00
		1.36E-12	1.38E+00
		1.10E-12	1.12E+00
		5.20E-13	5.27E-01
E	Measurements parallel to the bedding (bedded siltstone, marl, sandstone; Fig. 2D)	2.07E-14	2.09E-02
		1.46E-14	1.48E-02
		1.08E-14	1.09E-02
		6.24E-15	6.32E-03
		1.25E-14	1.27E-02
		1.29E-14	1.31E-02
		8.03E-15	8.13E-03
		7.95E-15	8.05E-03
		7.44E-15	7.54E-03
		1.31E-14	1.33E-02
		1.02E-14	1.03E-02
	Measurements orthogonal to the bedding (siltstone, marl; Fig. 2D)	6.16E-13	6.25E-01
		8.88E-14	8.99E-02
		2.71E-14	2.75E-02
		4.42E-12	4.48E+00
		3.32E-14	3.36E-02
		4.61E-14	4.67E-02
		6.30E-14	6.39E-02

		2.15E-14	2.18E-02
		2.30E-14	2.33E-02
		1.94E-13	1.97E-01
		1.27E-13	1.29E-01
F	Measurements orthogonal to a fault plane within foliated violet/blackish gouge	1.18E-14	1.20E-02
		1.39E-14	1.41E-02
		8.18E-15	8.28E-03
		2.00E-14	2.03E-02
		7.31E-15	7.40E-03
		9.07E-15	9.19E-03
		7.05E-15	7.15E-03
		9.92E-15	1.01E-02
		1.56E-14	1.58E-02
		7.15E-15	7.25E-03
G	Measurements parallel to the foliation (black gouge; Fig. 2E)	5.70E-13	5.77E-01
		8.00E-13	8.11E-01
		5.44E-13	5.52E-01
		6.71E-13	6.80E-01
		3.75E-13	3.80E-01
		1.09E-12	1.10E+00
		1.93E-13	1.95E-01
		1.18E-12	1.19E+00
		6.62E-13	6.70E-01
		1.68E-12	1.70E+00
		1.67E-12	1.70E+00
		6.42E-13	6.50E-01
		2.68E-13	2.71E-01
		4.22E-13	4.27E-01
		1.31E-12	1.33E+00

Supplementary Table 2. XRD data. Qz–quartz, Ab–albite, Dol–dolomite, Ank–ankerite, Py–pyrite, Hem–hematite, Rt–rutile, Pg–paragonite, Ms-$2M_1$ d/n–muscovite $2M_1$ polytype detrital/neoformed, Chl–chlorite, Kln–kaolinite, I-S–mixed-layers illite-smectite; R–stacking order of I-S, %I in I-S–percentage of illite in I-S.

Sample ID	Grain-size fractions (μm)	Mineral assemblage (wt.%)												R	%I in I-S
		Qz	Ab	Dol	Ank	Py	Hem	Rt	Pg	Ms-$2M_1$ d/n	Chl	Kln	I-S		
RGA K-Ar1	6-10	33	-	3	-	-	2	1	2	49	2	8	-		
	2-6	21	-	2	-	-	1	1	3	61	3	8	-		
	0.4-2	3	-	1	-	-	1	1	4	46	3	11	30	0	30
	0.1-0.4	-	-	-	-	-	-	-	-	50	10	-	40		
	<0.1	-	-	-	-	-	-	-	-	37	13	-	50		
RGA K-Ar2	6-10	47	-	5	-	-	7	2	5	25	8	1	-		
	2-6	39	-	4	-	-	5	2	6	34	8	2	-		
	0.4-2	5	-	-	-	-	3	1	4	49	9	2	27	1	80
	0.1-0.4	-	-	-	-	-	-	-	-	39	10	1	50		
	<0.1	-	-	-	-	-	-	-	-	28	5	-	67		
RGA K-Ar4	6-10	56	-	-	2	1	4	2	2	23	9	1	-		
	2-6	44	-	-	1	-	2	2	3	35	11	2	-		
	0.4-2	5	-	-	-	-	1	1	5	54	7	2	25	0	30
	0.1-0.4	-	-	-	-	-	-	-	-	61	9	-	30		
	<0.1	-	-	-	-	-	-	-	-	61	6	-	33		
RGA K-Ar5	6-10	34	3	-	-	-	1	1	1	44	16	-	-		
	2-6	26	3	-	-	-	-	1	2	51	17	-	-		
	0.4-2	6	1	-	-	-	-	1	-	40	23	-	29	1	80
	0.1-0.4	-	-	-	-	-	-	-	-	36	12	-	52		
	<0.1	-	-	-	-	-	-	-	-	30	10	-	60		
RGA K-Ar6	6-10	34	-	-	-	-	7	1	-	36/6	16	-	-		
	2-6	12	-	-	-	-	2	2	-	46/18	20	-	-		
	0.4-2	3	-	-	-	-	2	1	-	23/61	10	-	-	-	-
	0.1-0.4	-	-	-	-	-	1	-	-	23/70	6	-	-		
	<0.1	-	-	-	-	-	-	-	-	6/89	5	-	-		

Supplementary Table 3. K-Ar data. Spreadsheet with K-Ar data of the five separated fractions for the CF fault gouges.

			$^{40}Ar^{*}$				K			Age Data	
Outcrop	Sample Name	Fraction	Mass mg	mol/g	σ (%)	$^{40}Ar^{*}$ %	Mass mg	wt %	σ (%)	Age (Ma)	σ (Ma)
RGA	RGAK-Ar1	<0.1	7.158	1.646E-10	0.39	34.5	53.4	4.93	1.5	19.2	±0.3
	RGAK-Ar1	0.1-0.4	3.488	1.905E-10	0.46	38.5	49.9	4.67	1.5	23.4	±0.4
	RGAK-Ar1	0.4-2	2.390	2.117E-10	0.53	42.5	51.9	4.48	1.5	27.0	±0.4
	RGAK-Ar1	2-6	1.560	2.619E-10	0.64	66.6	54.2	3.94	1.6	37.9	±0.6
	RGAK-Ar1	6-10	2.088	2.571E-10	0.53	66.2	52.5	3.27	1.7	44.8	±0.8
	RGAK-Ar2	<0.1	2.284	2.024E-10	0.56	42.9	51.3	5.32	1.5	21.8	±0.3
	RGAK-Ar2	0.1-0.4	2.798	2.705E-10	0.45	53.6	50.6	5.31	1.5	29.1	±0.4
	RGAK-Ar2	0.4-2	2.476	3.272E-10	0.44	72.2	56.7	4.88	1.5	38.3	±0.6
	RGAK-Ar2	2-6	1.732	3.115E-10	0.53	86.5	55.3	2.64	1.7	66.9	±1.2
	RGAK-Ar2	6-10	2.504	2.628E-10	0.48	90.0	52.6	2.04	1.8	72.6	±1.3
	RGAK-Ar4	<0.1	2.256	1.621E-10	0.72	54.5	51.0	4.97	1.5	18.7	±0.3
	RGAK-Ar4	0.1-0.4	2.658	1.992E-10	0.58	58.4	58.2	4.91	1.4	23.2	±0.4
	RGAK-Ar4	0.4-2	3.056	2.523E-10	0.44	61.2	53.2	4.58	1.5	31.5	±0.5
	RGAK-Ar4	2-6	2.432	2.859E-10	0.47	79.1	52.4	2.61	1.7	62.0	±1.1
	RGAK-Ar4	6-10	2.902	2.254E-10	0.47	82.3	51.4	1.81	1.8	70.4	±1.3
	RGAK-Ar5	<0.1	3.800	9.318E-11	0.88	44.2	51.2	4.72	1.5	11.4	±0.2
	RGAK-Ar5	0.1-0.4	4.144	1.108E-10	0.76	53.7	53.4	4.69	1.5	13.6	±0.2
	RGAK-Ar5	0.4-2	2.124	1.094E-10	1.37	48.9	57.6	4.48	1.5	14.0	±0.3
	RGAK-Ar5	2-6	2.044	1.065E-10	0.98	66.8	51.0	3.68	1.6	16.6	±0.3
	RGAK-Ar5	6-10	2.410	9.364E-11	0.94	65.8	51.1	3.31	1.7	16.2	±0.3
	RGAK-Ar6	<0.1	2.832	3.918E-10	0.41	70.5	50.5	6.77	1.3	33.1	±0.5
	RGAK-Ar6	<0.1	1.724	3.902E-10	0.51	71.2	50.5	6.76	1.3	33.0	±0.5
	RGAK-Ar6	0.1-0.4	3.458	5.191E-10	0.38	82.6	53.5	7.21	1.2	41.0	±0.5
	RGAK-Ar6	0.4-2	2.122	6.182E-10	0.42	87.4	51.5	7.24	1.3	48.6	±0.6
	RGAK-Ar6	2-6	1.890	9.181E-10	0.42	96.6	51.6	6.37	1.4	81.3	±1.1
	RGAK-Ar6	6-10	1.698	1.082E-09	0.43	96.2	51.3	5.14	1.5	117.4	±1.8

Supplementary References

1. Dewey, J. F. Helman, M. L. Turco, E. Hutton, D. H. W. & Knott, S. D. Kinematics of the western Mediterranean. *Geol. Soc. Spec. Publ.* **45,** 265-283 (1989). doi:10.1144/GSL.SP.1989.045.01.15
2. Jolivet, L. Augier, R. Robin, C. Suc, J. P. & Rouchy, J. M. Lithospheric-scale geodynamic context of the Messinian salinity crisis. *Sediment. Geol.* **188,** 9-33 (2006). doi:10.1016/j.sedgeo.2006.02.004
3. Behr, W. M. & Platt, J. P. Kinematic and thermal evolution during two-stage exhumation of a Mediterranean subduction complex. *Tectonics* **31**(4) (2012). doi:10.1029/2012TC003121
4. Lonergan, L. & White, N. Origin of the Betic-Rif mountain belt. *Tectonics* **16,** 504-522 (1997). doi:10.1029/96TC03937
5. Vergés, J. & Fernàndez, M. Tethys–Atlantic interaction along the Iberia–Africa plate boundary: The Betic–Rif orogenic system. *Tectonophysics* **579,** 144-172 (2012). doi:10.1016/j.tecto.2012.08.032
6. Seber, D. Barazangi, M. Ibenbrahim, A. & Demnati, A. Geophysical evidence for lithospheric delamination beneath the Alboran Sea and Rif–Betic mountains. *Nature* **379,** 785-790 (1996). doi:10.1038/379785a0
7. Duggen, S., Hoernle, K., van den Bogaard, P., & Harris, C. (2004). Magmatic evolution of the Alboran region: The role of subduction in forming the western Mediterranean and causing the Messinian Salinity Crisis. *Earth Planet. Sci. Lett.* **218**(1-2), 91-108. doi:10.1016/S0012-821X(03)00632-0
8. Meijninger, B. M. L. & Vissers, R. M. L. Miocene extensional basin development in the Betic Cordillera, SE Spain revealed through analysis of the Alhama de Murcia and Crevillente Faults. *Basin Res.* **18,** 547-571 (2006). doi:10.1111/j.1365-2117.2006.00308.x
9. Palano, M. González, P. J. & Fernández, J. The Diffuse Plate boundary of Nubia and Iberia in the Western Mediterranean: Crustal deformation evidence for viscous coupling and fragmented lithosphere. *Earth Planet. Sci. Lett.* **430,** 439-447 (2015). doi:10.1016/j.epsl.2015.08.040
10. Spakman, W. Chertova, M. V. van den Berg, A. & van Hinsbergen, D. J. J. Puzzling features of western Mediterranean tectonics explained by slab dragging. *Nat. Geosci.* **11,** 211-216 (2018). doi:10.1038/s41561-018-0066-z
11. Keller, J. V. A. Hall, S. H. Dart, C. J. & McClay, K. R. The geometry and evolution of a transpressional strike-slip system: the Carboneras fault, SE Spain. *J. Geol. Soc. London* **152,** 339-351 (1995). doi:10.1144/gsjgs.152.2.0339
12. Rutter, E. H. Faulkner, D. R. & Burgess, R. Structure and geological history of the Carboneras Fault Zone, SE Spain: Part of a stretching transform fault system. *J. Struct. Geol.* **45,** 68-86 (2012). doi:10.1016/j.jsg.2012.08.009
13. Moreno X. et al. Quaternary tectonic activity of the Carboneras Fault in the La Serrata range (SE Iberia): Geomorphological and chronological constraints. *Tectonophysics* **663**, 78-94 (2015). doi:10.1016/j.tecto.2015.08.016
14. Boorsma, L. J. Syn-tectonic sedimentation in a Neogene strike-slip basin containing a stacked Gilbert-type delta (SE Spain). *Sediment. Geol.* **81,** 105-123 (1992). doi:10.1016/0037-0738(92)90059-Z

15. Martín-Algarra A. et al. Paleozoic Basement and Pre-Alpine History of the Betic Cordillera. in (eds. Quesada C. & Oliveira, J. T. The Geology of Iberia: A Geodynamic Approach, Regional Geology Reviews, vol. 2) 261-305 (Springer, 2019). doi:10.1007/978-3-030-10519-8_9
16. Giaconia F. et al. Compressional tectonic inversion of the Algero-Balearic basin: Latemost Miocene to present oblique convergence at the Palomares margin (Western Mediterranean), *Tectonics* **34,** 1516-1543 (2015). doi:10.1002/2015TC003861
17. Filomena, C. M. Hornung, J. & Stollhofen, H. Assessing accuracy of gas-driven permeability measurements: a comparative study of diverse Hassler-cell and probe permeameter devices. *Solid Earth* **5,** 1-11 (2014). doi:10.5194/se-5-1-2014
18. Fossen, H. Schultz, R. A. & Torabi, A. Conditions and implications for compaction band formation in the Navajo Sandstone, Utah. *J. Struct. Geol.* **33,** 1477-1490 (2011). doi: 10.1016/j.jsg.2011.08.001
19. R Core Team. R: A language and environment for statistical computing. R Foundation for Statistical Computing, Vienna, Austria (2024). url:https://www.R-project.org/
20. Ufer, K. Kleeberg, R. Bergmann, J. & Dohrmann, R. Rietveld refinement of disordered illite-smectite mixed-layer structures by a recursive algorithm. I: One-dimensional patterns. *Clays Clay Miner.* **60,** 507-534 (2012). doi:10.1346/CCMN.2012.0600507
21. Clauer, N., Zwingmann, H., Liewig, N., & Wendling, R. (2012). Comparative 40Ar/39Ar and K–Ar dating of illite-type clay minerals: A tentative explanation for age identities and differences. *Earth-Sci. Rev.* **115,** 76-96. doi:10.1016/j.earscirev.2012.07.003
22. Bauer, A., Velde, B., & Gaupp, R. (2000). Experimental constraints on illite crystal morphology. *Clay Miner.* **35,** 587-597. doi:10.1180/000985500546909
23. Doebelin, N. & Kleeberg, R. Profex: a graphical user interface for the Rietveld refinement program BGMN. *J. Appl. Cryst.* **48,** 1573-1580 (2015). doi:10.1107/S1600576715014685
24. Vermeesch, P. IsoplotR: A free and open toolbox for geochronology. *Geosci. Front.* **9,** 1479-1493 (2018). doi:10.1016/j.gsf.2018.04.001.
